# Longitudinal Trajectories of Body Roundness Index and Risk of Incident Hypertension in Non-Diabetic Elderly Adults: A Prospective Nationwide Cohort Study Involving Two Cohorts

**DOI:** 10.5334/gh.1587

**Published:** 2026-09-25

**Authors:** Xueping Gao, Yimou Liu, Bo Xiong, Yuan Xu, Jing Huang, Qiaoqiao Li

**Affiliations:** 1 Department of Geriatrics, The Second Affiliated Hospital of Chongqing Medical University, No. 76 Linjiang Road, Chongqing, 400010, China; 2 Department of Laboratory Medicine, The First Affiliated Hospital of Chongqing Medical University, Chongqing, 400042, China; 3 Chongqing Geriatric Specialized Nursing Clinical Diagnosis and Treatment Center (The Second Affiliated Hospital of Chongqing Medical University), Chongqing, China; 4 Department of Cardiology, The Second Affiliated Hospital of Chongqing Medical University, No. 76 Linjiang Road, Chongqing, 400010, China; 5 Department of Pathology, Zhongshan Hospital Fudan University, Shanghai, 200000, China

**Keywords:** body roundness index, hypertension, trajectory analysis, stacking ensemble machine learning, Shapley additive explanations

## Abstract

**Background:**

Central adiposity is a key determinant of hypertension, yet most studies rely on single-time-point measures or include individuals with diabetes, potentially confounding associations. The relationship between long-term body roundness index (BRI) trajectories and incident hypertension in non-diabetic populations remains unclear.

**Methods:**

We conducted a longitudinal analysis of non-diabetic middle-aged and older adults from the China Health and Retirement Longitudinal Study (CHARLS), with external validation using the English Longitudinal Study of Ageing (ELSA). In CHARLS, BRI measurements from Waves 1–3 (2011–2016) were used to construct trajectories, and incident hypertension was assessed at Wave 4 (2018–2020). In ELSA, trajectories were constructed using BRI measurements from Waves 2, 4, and 6 (2004–2013), and incident hypertension was assessed at Wave 8 (2016–2017). Associations were examined using multivariable logistic regression, with subgroup and sensitivity analyses. Machine learning models were additionally developed for multivariable hypertension risk prediction.

**Results:**

Three BRI trajectories were identified in both cohorts: low-level inverted U-shaped, moderate-level inverted U-shaped, and high-level increasing trajectories. Higher BRI trajectories were associated with a graded increase in incident hypertension risk in CHARLS. Compared with the low-level inverted U-shaped trajectory, the adjusted odds ratios (95% confidence intervals) were 1.40 (1.13–1.73) for the moderate-level inverted U-shaped trajectory and 1.76 (1.21–2.56) for the high-level increasing trajectory. Directionally consistent findings were observed in ELSA, particularly for the high-level increasing trajectory. Findings remained robust in subgroup and sensitivity analyses. Machine learning analyses showed that BRI trajectory provided complementary predictive information when combined with clinical and biomarker variables.

**Conclusions:**

Longitudinal BRI patterns were associated with incident hypertension in non-diabetic adults. Repeated assessment of BRI may provide complementary information for identifying individuals at elevated hypertension risk, although its causal and clinical implications require further investigation.

## Clinical Perspective

### What is new?

This study demonstrates that long-term BRI trajectories are independently associated with hypertension risk in non-diabetic adults across two large cohorts. Persistently high BRI trajectories confer the greatest risk. Incorporating BRI trajectories into machine learning models significantly enhances predictive performance and risk stratification. This study provides the first large-scale evidence for integrating longitudinal BRI trajectories with explainable machine learning for hypertension prediction.

### What are the clinical implications?

Longitudinal BRI monitoring may enable earlier and more precise identification of individuals at high risk of hypertension, supporting personalized prevention before overt metabolic disease develops.

**Figure:**
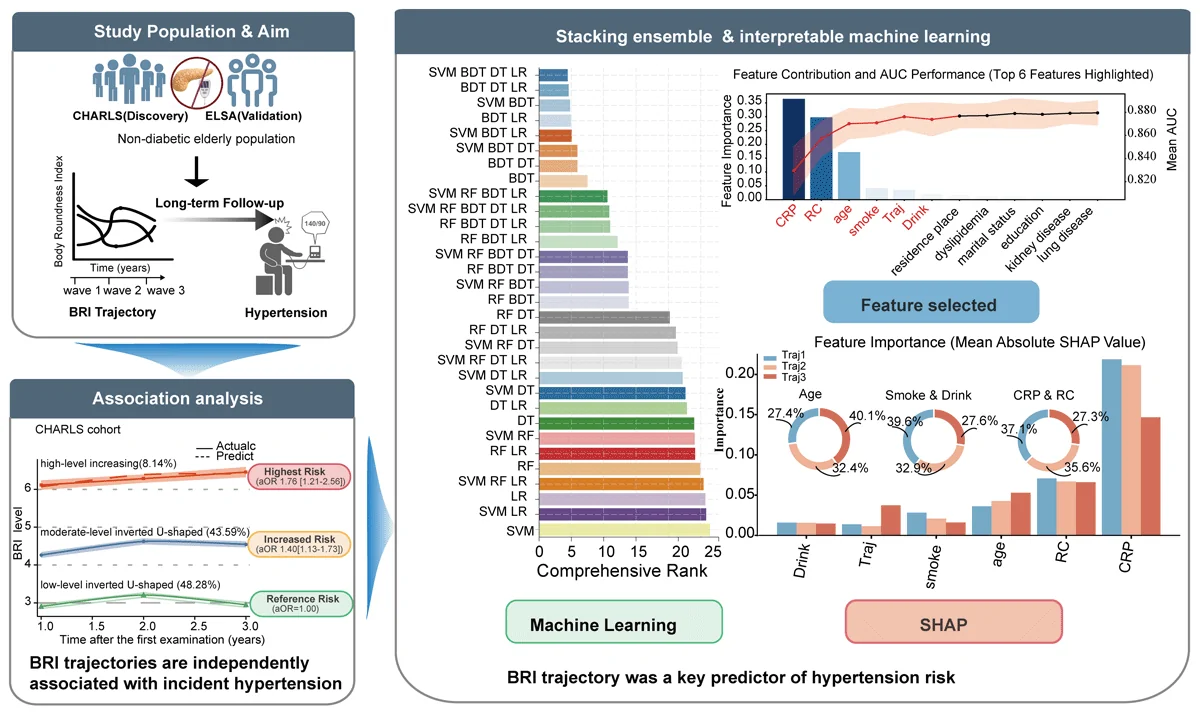


## Nonstandard Abbreviations and Acronyms

AUC: Area under the receiver operating characteristic curve
Avepp: Average posterior probability
BDT: Boosted decision tree
BMI: Body mass index
BRI: Body roundness index
CHARLS: China Health and Retirement Longitudinal Study
CI: Confidence interval
CRP: C-reactive protein
DBP: Diastolic blood pressure
DT: Decision tree
ELSA: English Longitudinal Study of Ageing
GBDT: Gradient boosting decision tree
GBTM: Group-based trajectory modeling
IPW: Inverse probability weighting
IRB: Institutional review board
Light-GBM: Light Gradient Boosting Machine
LOWESS: Locally weighted scatterplot smoothing
LR: Logistic regression
ML: Machine learning
NPV: Negative predictive value
OCC: Odds of correct classification
OR: Odds ratio
PPV: Positive predictive value
RC: Remnant cholesterol
RF: Random forest
ROC: Receiver operating characteristic
SBP: Systolic blood pressure
SHAP: Shapley Additive Explanations
SVM: Support vector machine
TG: Triglycerides
WC: Waist circumference
WHR: Waist-to-hip ratio
WHtR: Waist-to-height ratio

## Introduction

Hypertension remains a major global public health challenge and a leading modifiable risk factor for cardiovascular and renal diseases, contributing substantially to morbidity and mortality worldwide (1). Beyond its cardiovascular sequelae, hypertension is also recognized as an important modifiable risk factor for cognitive decline and dementia, underscoring the broad health impact of blood pressure control across the life course. With accelerated population aging, identifying early and actionable determinants of incident hypertension has become increasingly important (2). Notably, the residual lifetime risk of developing hypertension approaches 90% among adults who survive to older age, highlighting the near-ubiquity of hypertension risk in later life (3).

Obesity is a major upstream driver of primary hypertension, accounting for approximately 65–75% of the risk (4). In recent years, the obesity–hypertension link has been strengthened by converging evidence from large prospective cohorts, mechanistic studies, weight-loss interventions, and Mendelian randomization analyses, collectively supporting a causal role of excess adiposity and weight gain in elevating blood pressure and increasing the risk of incident hypertension (5,6). However, research focus has gradually shifted from general obesity indexed by body mass index (BMI) to body fat distribution, because central (abdominal/visceral) adiposity appears to confer greater cardiometabolic risk than BMI alone (7).

The body roundness index (BRI), proposed by Thomas et al. in 2013, is a geometry-based anthropometric indicator derived from waist circumference and height that aims to quantify body shape and central adiposity (8). Compared with conventional measures, BRI has been shown to better approximate total body fat percentage and visceral adipose tissue, offering a practical proxy for adiposity distribution using easily obtainable measurements. Such properties make BRI a promising and potentially more sensitive marker for identifying individuals at elevated cardiometabolic and hypertension risk.

Accumulating epidemiological evidence suggests that higher BRI is associated with prevalent and incident hypertension across populations (9,10). Diabetes may independently influence blood pressure through hyperglycemia, insulin resistance, vascular dysfunction, renal sodium retention, and glucose-lowering treatments. Including individuals with diabetes may therefore introduce metabolic and treatment-related heterogeneity and complicate the interpretation of the association between BRI and incident hypertension. Studies have found that individuals with type 2 diabetes have a higher prevalence of metabolic syndrome, which is associated with increased BRI (11). However, limited research has focused on whether changes in BRI are linked to incident hypertension in non-diabetic populations. This gap in the literature highlights the need for further investigation into the role of BRI in hypertension risk across different metabolic profiles, particularly in those without diabetes. In addition, much of the existing literature relies on cross-sectional designs or geographically restricted cohorts and, critically, uses a single baseline BRI measurement, which may not capture long-term exposure patterns (12,13). In aging populations, body composition and fat distribution change dynamically; therefore, longitudinal trajectories of BRI may better characterize cumulative and time-varying adiposity burdens and provide additional prognostic information beyond one-time measurements. Evidence from trajectory-based studies of obesity-related indices supports the clinical relevance of dynamic adiposity patterns for hypertension risk.

To address these gaps, this study focused on non-diabetic adults and used repeated BRI measurements to characterize longitudinal central adiposity patterns rather than relying on a single baseline value. We aimed to identify distinct BRI trajectories in the China Health and Retirement Longitudinal Study (CHARLS), examine their associations with incident hypertension, and externally validate the findings using the English Longitudinal Study of Ageing (ELSA). As a complementary analysis, we further evaluated whether BRI trajectory information could be integrated with routinely available clinical variables in machine learning (ML) models for incident hypertension risk prediction.

## Methods

### Study design and population

**Figure 1:**
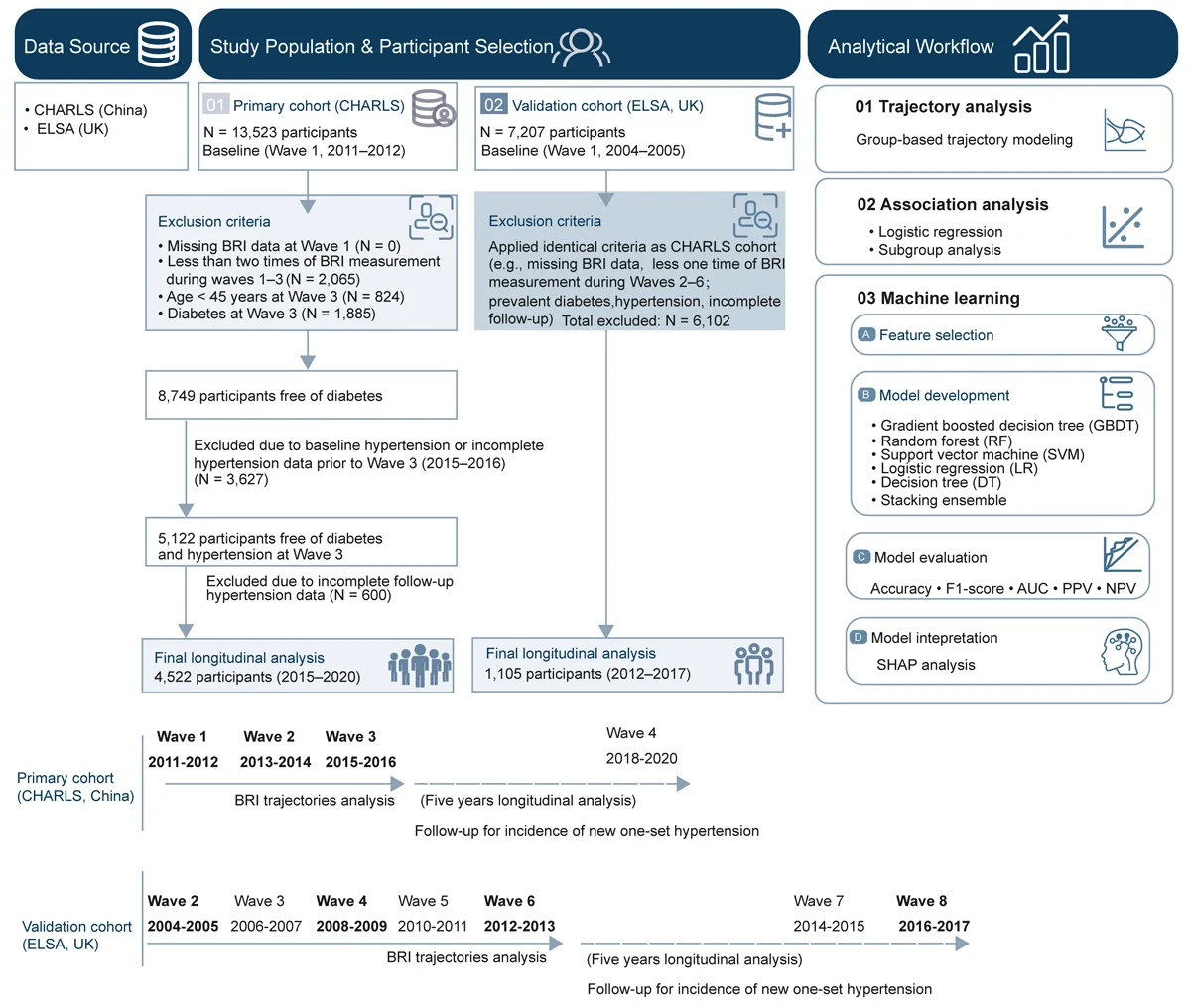
Study design, participant selection, and analytical workflow.

This study used data from CHARLS (14), a nationally representative, longitudinal survey of community-dwelling Chinese adults aged ≥45 years. CHARLS collects comprehensive information on sociodemographic characteristics, socioeconomic status, health behaviors, and biological markers. The baseline survey (Wave 1, 2011–2012) recruited 13,523 participants from 10,257 households across 28 provinces. Follow-up assessments were conducted biennially using face-to-face computer-assisted personal interviews, with blood samples collected in alternate waves. For the present analysis, data were obtained from Wave 1 and four subsequent follow-ups: Wave 2 (2013–2014), Wave 3 (2015–2016), and Wave 4 (2018–2020). Participant selection is shown in Figure 1. Participants were excluded if they had any of the following: (1) incomplete baseline nurse visit data required for body roundness index (BRI) assessment; (2) fewer than two visits with available BRI measurements; (3) age <45 years or a diagnosis of diabetes before follow-up; (4) prevalent hypertension before follow-up; or (5) incomplete follow-up information for incident hypertension. Participants were required to have valid BRI measurements at a minimum of two of the three trajectory-assessment waves. BRI measurements from Wave 1 (2011–2012), Wave 2 (2013–2014), and Wave 3 (2015–2016) were used to construct BRI trajectories, and incident hypertension was subsequently assessed at Wave 4 (2018–2020). A total of 4,522 participants was ultimately included in the analysis examining the association between BRI trajectories and incident hypertension. Ethical approval for CHARLS was obtained from the Biomedical Ethics Review Committee of Peking University (IRB 00001052-11015).

To externally validate the findings, data from ELSA (15) were used. ELSA is a nationally representative, prospective cohort study of community-dwelling adults aged ≥50 years in England. BRI data were available in Wave 2 (2004–2005), Wave 4 (2008–2009), Wave 6 (2012–2013), and Wave 8 (2016–2017). Consistent with the requirements of trajectory modeling in the CHARLS cohort, BRI measurements from Wave 2, Wave 4, and Wave 6 were used to construct BRI trajectories, with follow-up for incident hypertension until Wave 8. Except for the age criterion (≥50 years), the inclusion and exclusion criteria were consistent with those applied in the CHARLS cohort. A total of 1,105 participants was included in the validation analysis. Ethical approval for the ELSA study was granted by the London Multi-Centre Research Ethics Committee (MREC/01/2/91). All participants in both cohorts provided written informed consent. This study followed the Strengthening the Reporting of Observational Studies in Epidemiology (STROBE) reporting guidelines (16).

### Assessment of body roundness index and trajectory modeling

The body roundness index was calculated using the formula proposed by Thomas et al. (8). $\text{BRI}=364.2-365.5\times \sqrt{1-{\left(\frac{WC}{2\pi }\right)}^{2}/{\left(0.5\times \text{Height}\right)}^{2}}$ where WC (waist circumference) and height are measured in meters. To characterize the dynamic patterns of BRI over time, group-based trajectory modeling (GBTM) (17) was applied using the PROC TRAJ procedure in SAS software. In the CHARLS cohort, BRI measurements from Waves 1–3 (2011–2016) were used to construct longitudinal trajectories, and incident hypertension was subsequently assessed at Wave 4 (2018–2020). In the ELSA cohort, BRI measurements from Waves 2, 4, and 6 (2004–2013) were used for trajectory construction, and incident hypertension was assessed at Wave 8 (2016–2017). We adhered to principles of model parsimony and clinical interpretability. Models with two to four groups were fitted, testing linear, quadratic, and cubic functional forms for each group.

Model selection was guided by the following criteria: (1) minimization of the Bayesian Information Criterion (BIC); (2) adequate sample size per group (not less than 5% of the total population); (3) average posterior probability (AvePP) of assignment >0.70; (4) odds of correct classification (OCC) > 5; and (5) distinct visual separation of trajectories conforming to clinical plausibility.

### Outcome definition and covariates

The outcome was incident hypertension, assessed at Wave 4 (2018–2020) in CHARLS and at Wave 8 (2016–2017) in ELSA among participants without hypertension during the corresponding trajectory-assessment period. Hypertension was defined as a systolic blood pressure (SBP) ≥ 140 mmHg and/or a diastolic blood pressure (DBP) ≥ 90 mmHg, current use of antihypertensive medication, or a self-reported physician diagnosis of hypertension (based on the question, ‘Have you been diagnosed with hypertension by a doctor?’). The diagnostic criteria for hypertension in the ELSA cohort were identical to those applied in the CHARLS cohort.

Covariates assessed from Waves 1–4 in the CHARLS cohort included the following: (1) sociodemographic characteristics (age, sex, education level, and residence); (2) lifestyle behaviors (smoking status and alcohol consumption); (3) health status (history of cardiovascular and metabolic diseases); and (4) biomarkers (C-reactive protein, triglycerides, and remnant cholesterol). Continuous covariates were summarized using their mean values across the exposure period, where appropriate. Education level was categorized as below high school or high school and above. Residence was classified as rural or urban. Marital status was categorized as married or living with a stable partner versus other statuses (divorced, separated, widowed, or single). Alcohol consumption was defined as drinking alcohol at least once per week. Dyslipidemia, lung disease, kidney disease, and heart disease were identified based on self-reported physician diagnoses. We imputed the missing value for further exploration using the K Nearest Neighbors imputation method.

### Statistical analysis

Baseline characteristics were compared across BRI trajectory groups using analysis of variance (ANOVA) or Kruskal–Wallis tests for continuous variables, and χ² tests for categorical variables. Multivariable logistic regression models were employed to estimate odds ratios (ORs) and 95% confidence intervals (CIs) for the association between BRI trajectories and incident hypertension. As a separate but complementary predictive analysis, a structured ML framework was implemented to assess whether BRI trajectory information could be integrated with clinical variables for multivariable hypertension risk prediction. Prior to model development, correlation analyses were conducted among all clinical variables. For pairs of variables with a correlation coefficient greater than 0.6, only one variable was retained to reduce multicollinearity among the independent variables in the models. Subsequently, feature selection was performed using a Light Gradient Boosting Machine (Light-GBM (18,19)) to identify the most informative predictors based on feature importance rankings. Specifically, feature importance was first quantified using the Light-GBM model, and variables were ranked accordingly. Sequential forward feature selection was then performed by iteratively adding variables in order of importance until the inclusion of additional features resulted in an area under the receiver operating characteristic curve (AUC) increase of less than 0.1. The selected feature set was used for final model construction (20). Model performance was evaluated using 10-fold cross-validation for each candidate feature set, with the mean receiver operating characteristic (ROC) AUC calculated across folds. The 95% confidence intervals were estimated based on 10 fold-specific results and visualized to illustrate model performance uncertainty. To balance the positive and negative samples, the training set underwent preprocessing with random under-sampling and synthetic minority over-sampling technique (SMOTE) over-sampling. Subsequently, several ML algorithms were developed (21), including logistic regression (LR (22)), decision tree (DT (23)), random forest (RF (24)), support vector machine (SVM (25)), and gradient boosting decision tree (GBDT (26)). In addition, a stacking ensemble model (27) was constructed by combining SVM, GBDT, RF, and DT as base learners, with logistic regression serving as the meta-learner to integrate predictions from individual models (28,29,30). The stacking ensemble model was used to improve predictive performance by combining multiple heterogeneous base learners, thereby reducing model-specific bias and variance and enhancing generalization ability. The dataset was randomly split into a training set (70%) and a testing set (30%). Model training and hyperparameter tuning were conducted within the training set using 10-fold cross-validation. Model performance was evaluated in the testing set using multiple metrics, including accuracy, sensitivity, specificity, F1-score, area under the receiver operating characteristic curve, positive predictive value (PPV), and negative predictive value (NPV). To enhance model interpretability, Shapley Additive Explanations (SHAP) (31) were applied, particularly for tree-based models (29,32). Mean absolute SHAP values were used to quantify global feature importance, while SHAP summary plots illustrated the direction and magnitude of each predictor’s contribution to hypertension risk. Furthermore, SHAP dependence plots with locally weighted regression (LOWESS) smoothing were used to explore potential non-linear relationships between key predictors and SHAP value.

Sensitivity analyses were conducted to examine the robustness of the main findings in this prospective cohort study. First, analyses were restricted to participants with complete data for all covariates. Second, the association between baseline BRI and the incidence of hypertension during follow-up was re-evaluated. Third, participants who were using lipid-lowering medications at baseline were excluded. Fourth, participants with a history of cancer at baseline were excluded. In addition, inverse probability weighting (IPW) logistic regression was applied to further reduce potential confounding bias. Furthermore, sensitivity analyses were conducted after excluding potential metabolic and clinical intermediates to re-evaluate the associations between BRI trajectory groups and incident hypertension. Finally, Cox regression was performed as a sensitivity analysis, with follow-up starting at Wave 3 after completion of BRI trajectory assessment. Participants with hypertension before or at Wave 3 were excluded, and follow-up continued until incident hypertension. All statistical analyses were performed using R software (version 4.5.2). Two-sided P values <0.05 were considered statistically significant.

## Results

### Baseline characteristics

**Figure 2:**
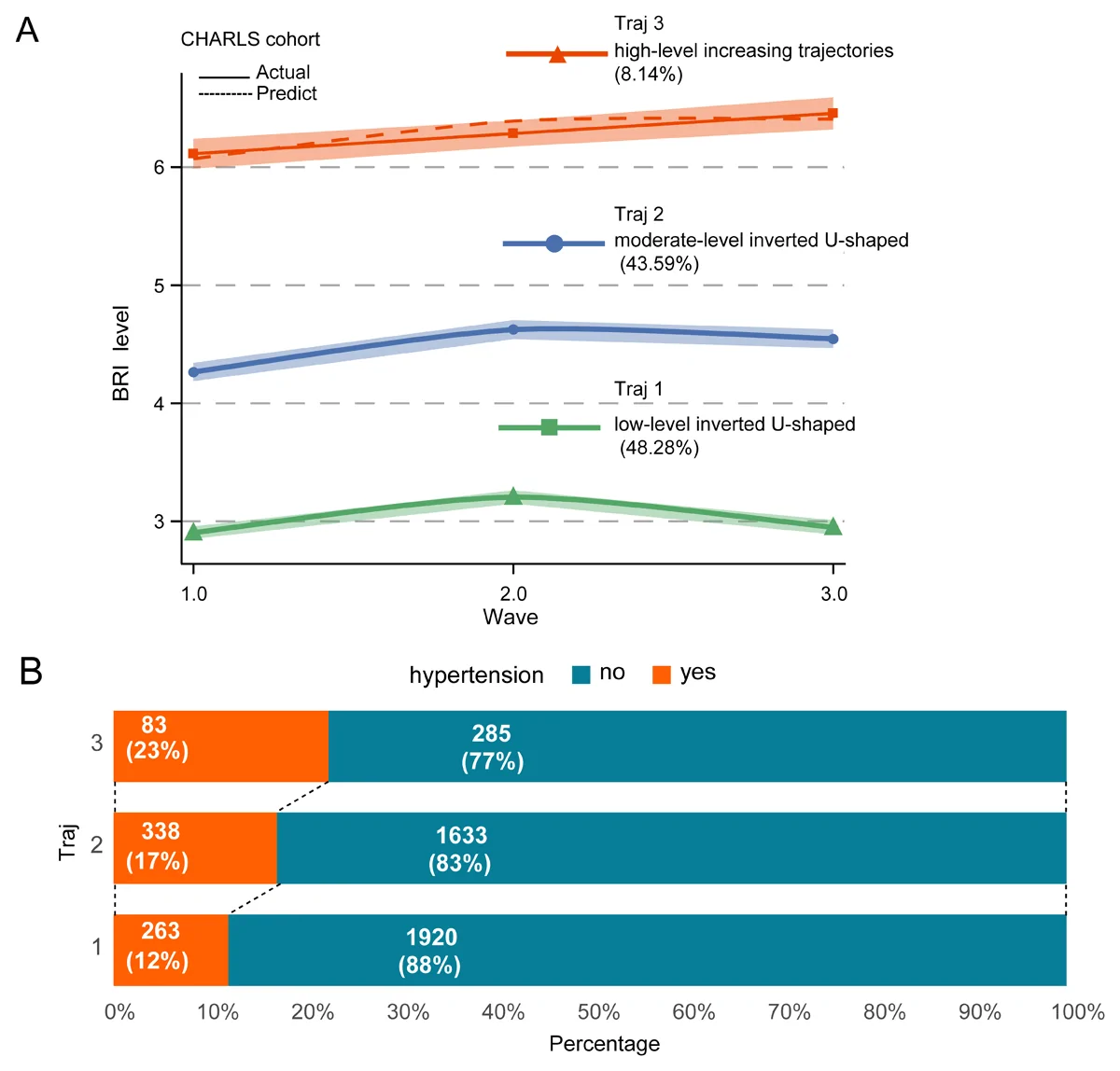
Longitudinal trajectories of body roundness index and incidence of hypertension in the CHARLS cohort. **(A)** Three-group BRI trajectory model. **(B)** Incidence of hypertension according to trajectory group.

A total of 4,522 participants from the CHARLS cohort were included in the final group-based trajectory modeling analysis. Linear, quadratic, and cubic trajectory models with two, three, and four groups were fitted, respectively. Model selection was based on a comprehensive evaluation of fit indices, including the Bayesian Information Criterion (BIC), Akaike Information Criterion (AIC), entropy, and average posterior probabilities, as well as the substantive interpretability of the trajectory shapes (Tables S1–S2). On the basis of these criteria, a three-group model, comprising two quadratic trajectories and one linear trajectory, was identified as the optimal model (Table 1). Three distinct BRI trajectories were identified: low-level inverted U-shaped (trajectory 1; n = 2,183), moderate-level inverted U-shaped (trajectory 2; n = 1,971), and high-level increasing (trajectory 3; n = 368) patterns (Figure 2A). The incidence of hypertension was 12%, 17%, and 23% in trajectories 1, 2, and 3, respectively (Figure 2B). Baseline clinical characteristics according to BRI trajectory groups are summarized in Table 2. The mean age of the study population was 60.29 ± 8.51 years. Significant differences in baseline characteristics were observed across BRI trajectory groups. Compared with participants in the low-level inverted U-shaped BRI trajectory, those in the high-level increasing trajectory had markedly higher body mass index and were more likely to be female and reside in urban areas. Participants in the high-level increasing BRI group also had lower proportions of current smoking and alcohol consumption. In addition, the baseline prevalence of dyslipidemia and heart disease was higher in the high-level increasing trajectory group. No significant differences were observed across trajectory groups with respect to marital status, education level, stroke, lung disease, cancer, or kidney disease.

**Table 1:** Evaluation index of optimal trajectory model.


TRAJECTORY GROUP	SHAPE	AVEPP%	n	(Pj)	(πj)	OCC	Ej

Group1	Quadratic	89.06	2183	48.28	47.91	8.8	0.732

Group2	Quadratic	85.23	1971	43.59	43.47	7.5	

Group3	Linear	87.17	368	8.14	8.62	72.0	


Avepp, average posterior probability; Pj, estimated group membership probability; πj, observed group proportion; OCC, odds of correct classification; Ej, classification error index.

**Table 2:** Baseline characteristics of the study population according to BRI trajectory.


VARIABLE	TOTAL (n = 4,522)	TRAJ 1 (n = 2,183)	TRAJ 2 (n = 1,971)	TRAJ 3 (n = 368)	STATISTIC	p-value*

**Age**	60.29 ± 8.51	60.59 ± 8.45	59.87 ± 8.54	60.81 ± 8.64	4.46	0.01

**Body mass index**	22.74 ± 3.28	20.80 ± 2.30	24.01 ± 2.57	27.51 ± 3.52	1536.34	<0.0001

**Sex**					405.82	<0.0001

female	2458(54.36)	868(39.76)	1282(65.04)	308(83.70)		

male	2064(45.64)	1315(60.24)	689(34.96)	60(16.30)		

**Marital status**					1.77	0.41

married	4022(88.94)	1955(89.56)	1744(88.48)	323(87.77)		

other	500(11.06)	228(10.44)	227(11.52)	45(12.23)		

**Residence place**					30.67	<0.0001

rural	3104(68.64)	1584(72.56)	1287(65.30)	233(63.32)		

urban	1418(31.36)	599(27.44)	684(34.70)	135(36.68)		

**Education**					3.17	0.20

High school or above	479(10.59)	234(10.72)	216(10.96)	29 (7.88)		

Middle school and below	4043(89.41)	1949(89.28)	1755(89.04)	339(92.12)		

**Drink**					120.82	<0.0001

no	2962(65.50)	1267(58.04)	1394(70.73)	301(81.79)		

yes	1560(34.50)	916(41.96)	577(29.27)	67(18.21)		

**Smoke**					335.23	<0.0001

Never	2692(59.53)	1026(47.00)	1353(68.65)	313(85.05)		

Former smoker	535(11.83)	290(13.28)	226(11.47)	19 (5.16)		

Current smoker	1295(28.64)	867(39.72)	392(19.89)	36 (9.78)		

**Stroke**					3.71	0.16

no	4458(98.58)	2151(98.53)	1948(98.83)	359(97.55)		

yes	64 (1.42)	32 (1.47)	23 (1.17)	9 (2.45)		

**Dyslipidemia**					120.91	<0.0001

no	3454(76.38)	1821(83.42)	1393(70.67)	240(65.22)		

yes	1068(23.62)	362(16.58)	578(29.33)	128(34.78)		

**Lung disease**					4.77	0.09

no	3941(87.15)	1878(86.03)	1739(88.23)	324(88.04)		

yes	581(12.85)	305(13.97)	232(11.77)	44(11.96)		

**Cancer**					0.11	0.95

no	4467(98.78)	2157(98.81)	1946(98.73)	364(98.91)		

yes	55 (1.22)	26 (1.19)	25 (1.27)	4 (1.09)		

**Kidney disease**					0.94	0.63

no	4114(90.98)	1977(90.56)	1802(91.43)	335(91.03)		

yes	408 (9.02)	206 (9.44)	169 (8.57)	33 (8.97)		

**Heart disease**					13.42	<0.01

no	4035(89.23)	1983(90.84)	1737(88.13)	315(85.60)		

yes	487(10.77)	200 (9.16)	234(11.87)	53(14.40)		

**Incident hypertension**					38.21	<0.0001

no	3838(84.87)	1920(87.95)	1633(82.85)	285(77.45)		

yes	684(15.13)	263(12.05)	338(17.15)	83(22.55)		


*ANOVA, the Kruskal-Wallis test, and the Chi-square test assume independence of observations. Traj, trajectory.

In the ELSA validation cohort, three BRI trajectories comparable to those identified in CHARLS were observed: low-level inverted U-shaped (trajectory 1; n = 486), moderate-level inverted U-shaped (trajectory 2; n = 513), and high-level increasing (trajectory 3; n = 106) patterns, with satisfactory model fit (Figure S1A). The corresponding incidences of hypertension were 17%, 19%, and 27%, respectively (Figure S1B), supporting the general consistency of the findings across cohorts.

### Associations between BRI trajectories and hypertension incidence

In the CHARLS cohort, higher BRI trajectories were associated with increased odds of incident hypertension, and the associations remained after sequential multivariable adjustment (Table 3). In the fully adjusted model, compared with the low-level inverted U-shaped trajectory, the ORs were 1.40 (95% CI, 1.13–1.73) for the moderate-level inverted U-shaped trajectory and 1.76 (95% CI, 1.21–2.56) for the high-level increasing trajectory, with a significant trend across trajectory groups (P for trend <0.001).

**Table 3:** Association between trajectories of BRI and new-onset hypertension risk.


CHARACTER	CRUDE MODEL		MODEL 1		MODEL 2		MODEL 3	
			
	*OR (95%CI)*	*P*	*OR (95%CI)*	*P*	*OR (95%CI)*	*P*	*OR (95%CI)*	*P*

Trajectory								

1	ref		ref		ref		ref	

2	1.51(1.27,1.80)	<0.0001	1.57(1.31,1.88)	<0.0001	1.39(1.13,1.72)	0.002	1.40(1.13,1.73)	0.002

3	2.13(1.61,2.80)	<0.0001	2.21(1.66,2.95)	<0.0001	1.77(1.22,2.56)	0.003	1.76(1.21,2.56)	0.003

p for trend		<0.0001		<0.0001		<0.001		<0.001


Crude model includes only the trajectory of BRI variable; Model 1 was adjusted for age, sex, marital status, residence, and education; Model 2 was additionally adjusted for c-reactive protein, triglycerides, remnant cholesterol, smoking, and alcohol consumption; Model 3 was further adjusted for dyslipidemia, heart disease, stroke, kidney disease, and lung disease. OR (95%CI), odds ratios and 95% confidence intervals; BRI, body roundness index.

In the ELSA cohort, the high-level increasing trajectory remained associated with increased odds of incident hypertension in the fully adjusted model (OR, 1.67; 95% CI, 1.01–2.75), whereas the association for the moderate-level inverted U-shaped trajectory was not statistically significant (Table 4). Although the trend across trajectory groups was attenuated after multivariable adjustment, the direction of the estimates was generally consistent with that observed in CHARLS. These results should be interpreted cautiously because the high-level trajectory group included only 106 participants.

**Table 4:** Association between BRI trajectory and hypertension incidence in ELSA cohort.


VARIABLE	CRUDE MODEL		MODEL 1		MODEL 2		MODEL 3	
			
	*OR (95%CI)*	*P*	*OR (95%CI)*	*P*	*OR (95%CI)*	*P*	*OR (95%CI)*	*P*

Trajectory								

1	ref		ref		ref		ref	

2	1.13(0.82,1.56)	0.461	1.05(0.76,1.47)	0.759	1.05(0.75,1.47)	0.764	1.04(0.74,1.45)	0.835

3	1.78(1.09,2.89)	0.021	1.67(1.02,2.74)	0.043	1.65(1.00,2.71)	0.049	1.67(1.01,2.75)	0.044

p for trend		0.042		0.101		0.109		0.112


Crude model includes only the trajectory of BRI variable; Model 1 was adjusted for age, sex, marital status, job, income and education; Model 2 was additionally adjusted for drink and smoking status; Model 3 was further adjusted for cancer, arrhythmia, and chronic obstructive pulmonary disease. BRI, body roundness index; OR (95%CI), odds ratios and 95% confidence intervals.

### Subgroup analysis

In subgroup analyses stratified by age, sex, education, smoking status, alcohol consumption, and major comorbidities, the positive association between higher BRI trajectories and incident hypertension remained generally consistent in both cohorts.

In the CHARLS cohort (Figure S2), participants in the high-level increasing BRI trajectory consistently exhibited significantly elevated risks of incident hypertension across nearly all subgroups, with odds ratios ranging from approximately 1.88 to 2.26 when compared with the low-level inverted U-shaped trajectory. A clear dose–response pattern across increasing BRI trajectories was observed in most strata (all P for trend <0.01). The magnitude of association appeared slightly stronger among older individuals (>60 years) and those without major comorbidities, although formal interaction tests did not indicate significant effect modification (all P for interaction >0.05).

In the ELSA cohort (Figure S3), the overall direction of associations was consistent with that observed in CHARLS. Participants in the high-level increasing BRI trajectory generally showed higher risks of hypertension across most subgroups; however, statistical significance was not uniformly achieved in certain strata, particularly among the oldest age group and participants with cancer, likely reflecting limited sample size and reduced statistical power.

Importantly, no statistically significant interactions were detected between BRI trajectories and any subgroup variables in either cohort, indicating that the association between long-term BRI patterns and hypertension risk was broadly stable across demographic and clinical subgroups.

### Machine learning analysis

#### Feature screening

**Figure 3:**
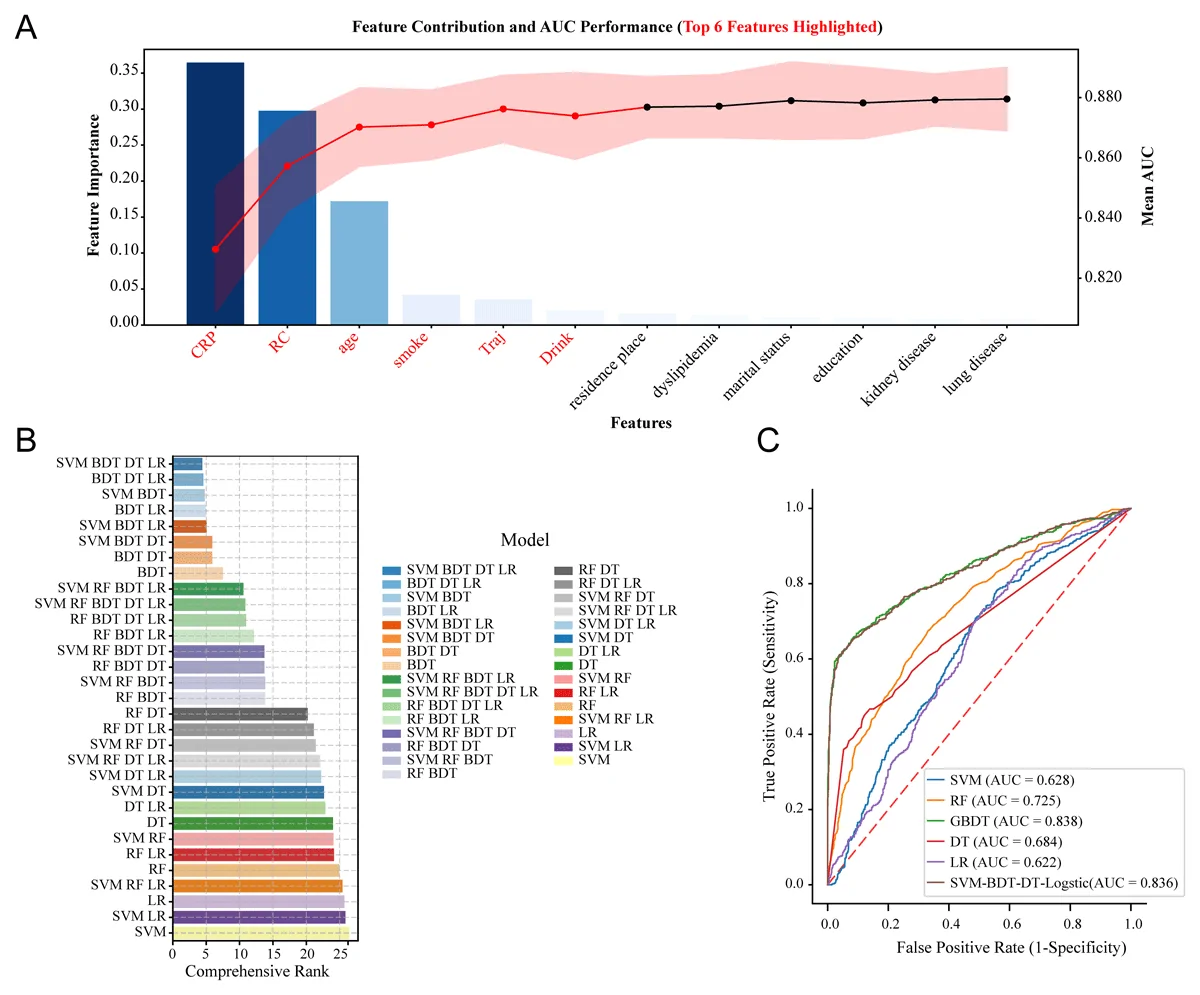
The comprehensive rank of five independent base ML models and various stacking ensemble combinations. **(A)** Features panel selection strategy implemented in the training set. Bars represent feature importance ranked by contribution to the prediction of incident hypertension. The line plot shows the cumulative area under the receiver operating characteristic curve (AUC; right y-axis) as features were sequentially added. Shaded areas indicate 95% confidence intervals estimated using 10-fold cross-validation. Features selected for the final model are highlighted in red and were identified based on the absence of significant incremental improvement in model performance over two consecutive iterations, as assessed by DeLong’s test (two-sided P > 0.05). **(B)** Comprehensive ranking of five individual machine learning models, random forest (RF), decision tree (DT), gradient boosting decision tree (GBDT), support vector machine (SVM), and logistic regression, and multiple stacking ensemble models in the testing set. The SVM–BDT–DT–Logistic stacking ensemble achieved the highest overall rank based on combined performance metrics. **(C)** Comparison of AUC values between the SVM–BDT–DT–Logistic stacking ensemble and individual machine learning models (RF, DT, GBDT, SVM, and logistic regression).

Feature selection was conducted using a two-step pipeline comprising multicollinearity assessment and Light Gradient Boosting Machine (Light-GBM)-based AUC-driven selection to optimize prediction of incident hypertension. First, correlation analyses identified highly collinear variable pairs (trajectory group and body mass index, smoking status and sex, remnant cholesterol and triglycerides; r > 0.64), from which one variable in each pair was retained. Consequently, 12 candidate variables, C-reactive protein (CRP), remnant cholesterol, age smoking and drinking status, trajectory group, residence, dyslipidemia, marital status, education level, kidney disease, and lung disease, were included for further analysis (Figure S4A), and their relative importance was ranked using Light-GBM (Figure S4B). Second, sequential forward selection based on incremental AUC improvement was applied to identify the most informative feature subset. A final panel of six features (CRP, remnant cholesterol (RC), age, smoke, alcohol consumption, and trajectory) was selected for model construction, with CRP, remnant cholesterol, and age emerging as the strongest predictors (AUC > 0.80). Inclusion of additional features further improved model performance, with the AUC approaching 0.88 (Figure 3A).

#### Single, stacking ensemble model development and evaluation

Five base models, including GBDT, RF, logistic regression, DT, and SVM, were evaluated as individual classifiers. In addition, 26 stacking ensemble model combinations were constructed and tested. In total, 31 models were assessed. The overall predictive performance of all individual models and stacking ensemble combinations is presented in Table S3.

#### Optimal predictive model selection

Our aim was to develop and compare multiple individual machine learning models and stacking ensemble models for early prediction of incident hypertension among 4,522 non-diabetic older adults and to identify the optimal approach. Models were ranked using a prespecified set of performance metrics, including accuracy, specificity, sensitivity, and F1 score. Based on this comprehensive evaluation, the five top-performing models were SVM–BDT–DT–LR, BDT–DT–LR, SVM–BDT, BDT–LR, and SVM–BDT–LR (Table S4). Notably, the SVM–BDT–DT–Logistic stacking ensemble demonstrated discriminative performance comparable to that of the best individual model, with an AUC of 0.836. Although the standalone GBDT model achieved a marginally higher AUC (0.838), the SVM–BDT–DT–Logistic ensemble consistently outperformed other models when evaluated across multiple prespecified performance metrics, including AUC, accuracy, F1 score, negative predictive value, positive predictive value, specificity, and sensitivity. Based on this comprehensive assessment, the SVM–BDT–DT–Logistic stacking ensemble was identified as the overall best-performing model (Figure 3B, C).

#### SHAP-based model interpretation and feature contribution patterns

**Figure 4:**
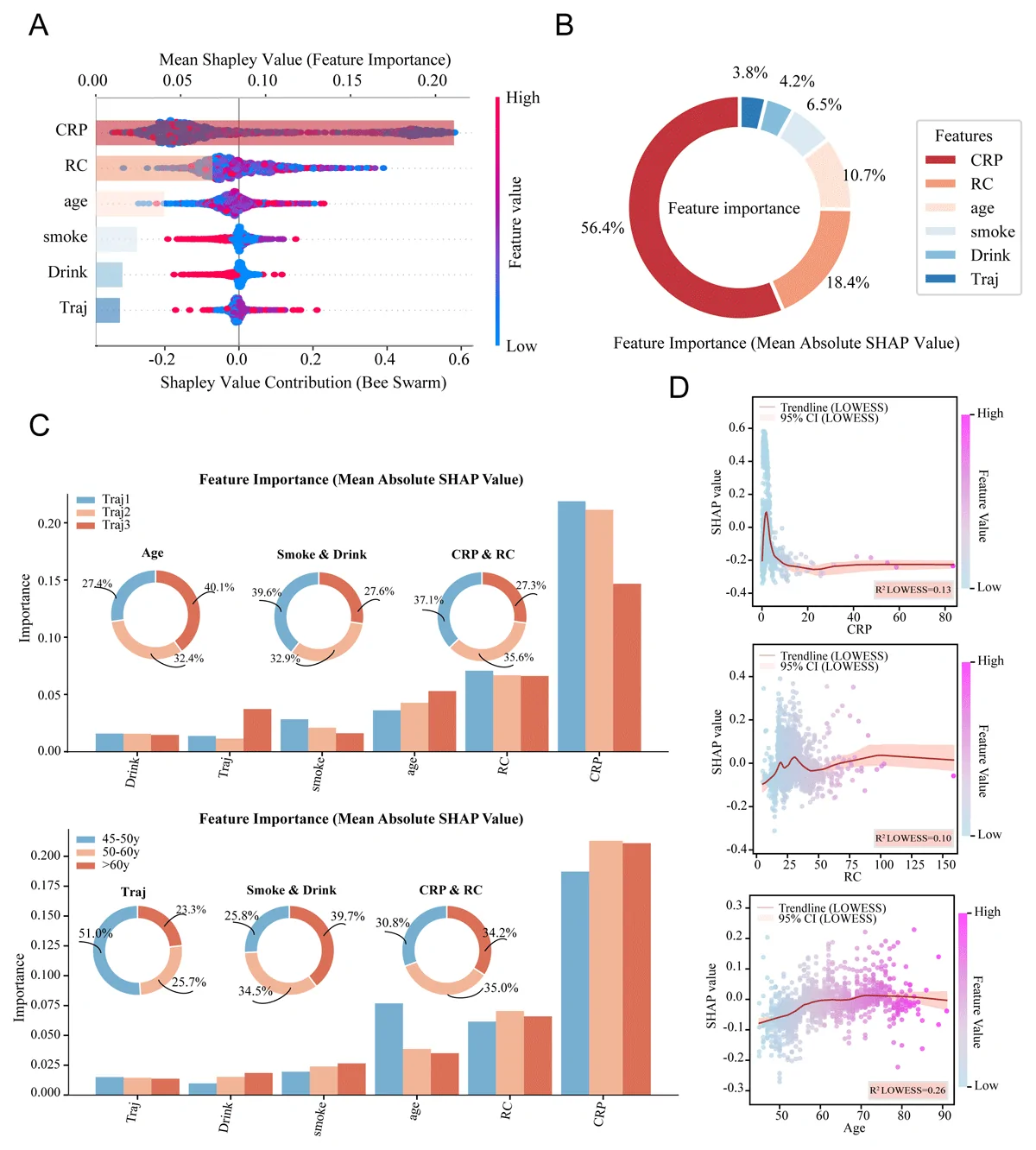
Global model interpretability analysis. (A) Combined SHAP summary and bar plots for the six features included in the hypertension risk prediction model. SHAP values represent the contribution of each feature to the model output. Each dot corresponds to an individual participant in the testing dataset and is colored according to the feature value, with vertical stacking indicating feature value density. The bar plot shows the mean absolute SHAP value for each feature. (B) Pie chart showing the relative percentage contribution of each of the six features to the prediction model, calculated based on the mean absolute SHAP values. (C) Bar plots and pie charts illustrating heterogeneity in feature contributions to the hypertension prediction model, quantified using SHAP values, across BRI trajectory groups (trajectory 1, trajectory 2, and trajectory 3) and age strata (45–50 years, 50–60 years, and >60 years). Feature groups include lifestyle factors (smoking and alcohol consumption) and biochemical markers (C-reactive protein and remnant cholesterol), with bars representing absolute contributions and pie charts indicating relative proportions. (D) SHAP dependence plots with locally weighted scatterplot smoothing (LOWESS) curves illustrating non-linear associations between continuous predictors (C-reactive protein, remnant cholesterol, and age) and model output. Each dot represents an individual participant, with SHAP values shown on the y-axis and corresponding feature values on the x-axis. The LOWESS curve summarizes the overall trend in feature contribution across the observed range of values.

Model interpretability was further assessed using SHAP. SHAP summary plots indicated that CRP and RC had the strongest overall influence on model predictions, with higher feature values generally contributing to an increased predicted risk of incident hypertension (Figure 4A). In addition, the proportional contribution analysis showed that CRP, RC, and age accounted for the largest shares of the total contribution among the six selected predictors, contributing 56.4%, 18.4%, and 10.7%, respectively, to the incident hypertension prediction model. In contrast, the BRI trajectory variable contributed the smallest proportion (3.84%) (Figure 4B). Stratified SHAP analyses demonstrated differential patterns of feature contributions across BRI trajectory and age subgroups. Across trajectory groups, the combined contribution of CRP and RC to the prediction model was higher in trajectory 1 (37.1%) and trajectory 2 (35.6%) than in trajectory 3 (27.3%). Across age strata, the relative contributions of CRP and RC were broadly comparable among participants aged 45–50 years (30.8%), 50–60 years (35.0%), and >60 years (34.2%). In contrast, the relative contributions of smoking and alcohol consumption increased with age, being higher in participants aged >60 years (39.7%) and 50–60 years (34.5%) compared with those aged 45–50 years (25.8%) (Figure 4C). SHAP dependence plots, complemented by LOWESS analyses, further suggested non-linear associations between key predictors and SHAP values of the corresponding predictors (Figure 4D).

### Sensitivity analyses

A series of sensitivity analyses were conducted to assess the robustness of the main findings (Table S5–S9). First, analyses restricted to participants without missing covariate values yielded results consistent with the primary analysis (Table S5). Second, when baseline BRI was analyzed instead of BRI trajectories, higher baseline BRI remained associated with incident hypertension. In the fully adjusted model, the OR was 1.24 (95% CI, 1.14–1.35) per 1-SD increase in baseline BRI, and participants in the highest tertile had an OR of 1.69 (95% CI, 1.36–2.10) compared with those in the lowest tertile (Table S6). To minimize potential confounding by medication use, additional analyses were performed after excluding participants who used lipid-lowering drugs, with results remaining largely unchanged (Table S7). Furthermore, exclusion of participants with cancer did not materially alter the observed associations (Table S8). We also used the inverse probability weighting method to adjust for measured confounders. Standardized mean differences (SMD) for the IPW-weighted covariates were calculated, and the results showed that SMD for all covariates was less than 10% after IPW adjustment (Figure S5). Weighted logistic regression analyses accounting for the complex survey design confirmed the positive association between BRI trajectories and hypertension risk (Table S9). In sensitivity analyses excluding potential metabolic and clinical intermediates, the associations between higher BRI trajectory groups and incident hypertension remained materially unchanged (Table S10). Finally, Cox proportional hazards regression yielded results consistent with those of the logistic regression models. Compared with trajectory 1, trajectory 2 and trajectory 3 were associated with higher risks of incident hypertension, with hazard ratios of 1.46 (95% CI, 1.23–1.72) and 1.92 (95% CI, 1.48–2.50), respectively. A significant dose–response trend was observed across BRI trajectory groups (P for trend = 0.01), supporting the robustness of the primary findings (Table S11).

## Discussion

In this longitudinal analysis of non-diabetic adults, we found that distinct trajectories of body roundness index were independently associated with the risk of incident hypertension. Compared with individuals in the low-level inverted U-shaped BRI trajectory, those with persistently moderate or high BRI trajectories exhibited a significantly higher risk of developing hypertension, even after comprehensive adjustment for sociodemographic factors, lifestyle behaviors, and cardiometabolic comorbidities. These associations were consistently observed across two independent cohorts and remained robust in multiple sensitivity analyses. Notably, the risk associated with BRI trajectories showed a graded pattern, with the highest risk observed among individuals with a persistently high BRI over time. Together, these findings highlight the importance of long-term patterns of central adiposity, rather than single-point anthropometric measurements, in the development of hypertension among individuals without diabetes. The body roundness index is considered a surrogate marker of central (abdominal) adiposity, which reflects preferential accumulation of visceral fat rather than generalized obesity. Central obesity is characterized by adipocyte hypertrophy and hyperplasia, which are accompanied by profound alterations in adipose tissue function. These changes include dysregulated secretion of adipokines, with reduced production of anti-inflammatory mediators and increased release of pro-inflammatory cytokines (33,34), as well as abnormal free fatty acid metabolism (35) and local tissue hypoxia (36). Such pathophysiological alterations may contribute to endothelial dysfunction, increased arterial stiffness, and impaired cardiovascular homeostasis, thereby substantially elevating the risk of hypertension and other cardiovascular diseases. From this perspective, BRI trajectories may capture not only long-term body shape patterns but also the cumulative burden of adverse adipose tissue-related biological processes that promote vascular dysfunction.

An important strength of the present study is the explicit focus on a non-diabetic population. Previous studies examining the association between adiposity-related indices and hypertension have frequently included individuals with diabetes or metabolically heterogeneous populations, in whom hyperglycemia, insulin resistance, and diabetes-related treatments may substantially influence blood pressure regulation (37,38,39,40). For example, an Iranian clinical cohort study of 1,685 normotensive patients with type 2 diabetes demonstrated that obesity-related indices, including body mass index, waist-to-hip ratio (WHR), and waist-to-height ratio (WHtR), were strong predictors of incident hypertension over a 4.8-year follow-up period (41). In such populations, however, dysglycemia, insulin resistance, and diabetes-related therapies may play a prominent role in shaping blood pressure trajectories.

By excluding participants with diabetes throughout the trajectory assessment period, our findings suggest that long-term central adiposity, as captured by BRI trajectories, is independently associated with the development of hypertension even in the absence of overt diabetes. This indicates that the observed association is unlikely to be driven solely by diabetic pathophysiology or glucose-lowering treatments, but rather reflects an earlier stage of cardiometabolic risk accumulation. From a clinical and public health perspective, these results extend existing evidence by demonstrating that elevated and persistent central adiposity confers excess hypertension risk before the onset of diabetes. This temporal separation supports the notion that hypertension may emerge earlier along the cardiometabolic continuum and underscores the potential value of identifying high-risk individuals based on longitudinal body shape patterns rather than established metabolic disease status. Together, these findings emphasize the relevance of monitoring central adiposity trajectories as part of early hypertension prevention strategies in metabolically non-diabetic populations.

Beyond the focus on a non-diabetic population, an additional strength of the present study lies in the use of longitudinal BRI trajectories rather than single baseline measurements. Trajectory-based analysis has increasingly been recognized as a powerful approach for capturing long-term exposure patterns and improving disease risk prediction. For example, Man Yang et al. demonstrated that higher longitudinal trajectories of body roundness index were significantly associated with an increased risk of cardiovascular disease, highlighting the prognostic value of dynamic body shape patterns beyond single-point assessments (42). Traditional epidemiological studies have predominantly relied on anthropometric indices assessed at a single time point, implicitly assuming that adiposity-related exposure remains stable over time (43,44,45). However, body composition and fat distribution are dynamic processes, particularly in middle-aged and older adults, and a single measurement may not adequately capture long-term exposure or cumulative metabolic burden. Prolonged exposure to elevated levels of obesity, especially central adiposity, may contribute to sustained increases in blood pressure through multiple interrelated pathways, including chronic activation of the renin–angiotensin–aldosterone system, heightened sympathetic nervous system activity, increased renal sodium reabsorption, endothelial dysfunction, and worsening insulin resistance (46). These mechanisms are inherently cumulative and time dependent, underscoring the limitations of cross-sectional anthropometric assessments.

In our study, trajectory-based modeling identified longitudinal BRI patterns characterized primarily by persistent low, moderate, and high levels, together with different within-group directions of change. Individuals in the high-level increasing trajectory had the greatest risk of incident hypertension after multivariable adjustment. Although baseline BRI was also associated with hypertension risk, trajectory analysis provided complementary information on the long-term level and temporal pattern of BRI exposure. From a methodological perspective, these findings suggest that longitudinal assessment of body shape may offer advantages over cross-sectional measures in characterizing cardiometabolic risk. Trajectory-based indicators are better positioned to reflect cumulative exposure to adverse adiposity-related pathways, such as sustained low-grade inflammation and long-term hemodynamic stress, which may not be fully captured by a single anthropometric measurement. Accordingly, incorporating longitudinal patterns of central adiposity may improve risk stratification for hypertension, particularly in populations without overt metabolic disease.

Interestingly, in the ELSA cohort, the associations between BRI trajectories and incident hypertension were not statistically significant among the oldest age group and participants with cancer. This finding should be interpreted with caution. First, these subgroups comprised relatively small sample sizes, particularly within the high-level increasing BRI trajectory, resulting in reduced statistical power and wider confidence intervals. Second, imbalance in the distribution of trajectory groups further limited the stability and precision of risk estimates. In addition, survivor bias may have occurred because individuals with both high adiposity and severe cardiometabolic risk are less likely to survive to advanced age or long-term cancer follow-up, leading to an underrepresentation of high-risk individuals in these subgroups. Moreover, the competing risk of mortality in older adults and cancer patients may preclude the occurrence or detection of hypertension during follow-up, thereby attenuating the observable association. Collectively, these methodological and statistical constraints suggest that the lack of statistical significance in these subgroups is more likely attributable to limited power and bias rather than a true absence of association.

The ML analyses provided complementary support for the main findings derived from traditional regression models (47). Across multiple algorithms, ensemble-based approaches demonstrated superior and robust predictive performance for incident hypertension, and key predictors consistently included BRI trajectory membership alongside age and inflammatory and metabolic markers. Importantly, the relative importance of BRI trajectories identified by the machine learning models was consistent with regression-based findings, highlighting the translational relevance of longitudinal central adiposity patterns for hypertension risk assessment. Focusing on a non-diabetic older population, we developed a hypertension risk prediction model that integrates BRI trajectory information with routinely collected clinical variables. This approach enables scalable and individualized risk stratification and has potential for integration into clinical and digital health decision-support systems to support early hypertension prevention (48). Explainable machine learning analyses using SHAP enhanced model interpretability. Higher BRI trajectories were associated with increased predicted hypertension risk, with consistent patterns across age and trajectory groups. SHAP dependence and LOWESS analyses further suggested non-linear relationships between key predictors and predicted model outputs, indicating potential threshold and saturation effects beyond linear modeling. These observations are consistent with the hypothesis that the influence of central adiposity on blood pressure regulation may accumulate over time and follow non-linear dynamics.

### Strengths

Several strengths of this study merit consideration. First, the use of longitudinal BRI trajectories allowed for characterization of long-term patterns of central adiposity, providing a more comprehensive assessment of cumulative exposure than single-point anthropometric measurements. Second, the restriction to a non-diabetic population minimized potential confounding from hyperglycemia, insulin resistance, and diabetes-related treatments, thereby clarifying the independent relationship between central adiposity trajectories and incident hypertension. Third, the consistency of findings across two independent cohorts, together with extensive sensitivity and subgroup analyses, supports the robustness and external validity of the results. Finally, the integration of traditional regression models with explainable ML approaches enhanced the interpretability and credibility of the observed associations.

### Limitations

Several limitations should be acknowledged. First, BRI was derived from anthropometric measurements rather than direct assessment of visceral adiposity, which may introduce measurement error. Second, residual confounding cannot be excluded, and the observational design precludes causal inference. Third, probabilistic trajectory assignment and the limited number of assessment waves may have introduced classification uncertainty and restricted the identification of more complex longitudinal patterns. Fourth, the machine learning analysis was exploratory and requires further external and prospective validation before clinical application. Finally, the findings may not be generalizable to younger populations or other ethnic groups.

## Conclusion

In conclusion, longitudinal BRI patterns were associated with incident hypertension among non-diabetic adults. Repeated assessment of BRI may provide complementary information beyond a single baseline measurement for hypertension risk assessment. However, given the observational design, these findings should not be interpreted causally, and further prospective studies are needed to evaluate their predictive and clinical utility.

## Additional File

The additional file for this article can be found as follows:

10.5334/gh.1587.s1 Supplementary Files. Tables S1 to S11 and Figures S1 to S5.

## Data Availability

All data are accessible at https://charls.pku.edu.cn/ (CHARLS) and https://www.elsa-project.ac.uk/ (ELSA).
