# Supplementary material for: Longitudinal Trajectories of Body Roundness Index and Risk of Incident Hypertension in Non-Diabetic Elderly Adults: A Prospective Nationwide Cohort Study Involving Two Cohorts
Source: Glob Heart. 2026 Sep 25;21(1):71. doi: 10.5334/gh.1587 (PMC13614250; doi:10.5334/gh.1587)
Supplement: Supplementary file 1 [file gh-21-1-1587-s1.pdf]

Additional File

Table S1 Model fit indices for selecting the optimal number of BRI trajectory groups.

Table S2 Parameter estimates of group-based trajectory models for BRI

Table S3 Overall performance of individual and stacking ensemble machine learning models for hypertension prediction.

Table S4 Comprehensive rank among different ML models.

Table S5 Association between BRI trajectory and hypertension incidence in a population excluding participants with any missing values

Table S6 Association between baseline BRI and hypertension incidence

Table S7 Association between BRI trajectory and hypertension incidence in a population after removing all participants who used lipid-lowering drugs

Table S8 Association between BRI trajectory and hypertension incidence in a population after removing all participants who has cancer

Table S9 Weighted logistic regression confirmed a positive association between BRI trajectory and hypertension risk

Table S10 Cox proportional hazards regression models confirmed a positive association between BRI trajectory and hypertension risk

Table S11 Weighted logistic regression confirmed a positive association between BRI trajectory and hypertension risk

Figure S1 Longitudinal trajectories of body roundness index and incidence of hypertension in the ELSA cohort

Figure S2 Subgroup analyses of the association between BRI trajectories and incident hypertension in the CHARLS cohort

Figure S3 Subgroup analyses of the association between BRI trajectories and incident hypertension in the ELSA cohort

Figure S4 Feature selection and importance ranking for hypertension prediction using Light-GBM

Figure S5 Differences in participant characteristics before and after weighting using the IPW method

Table S1 Model fit indices for selecting the optimal number of BRI trajectory groups

**Model**	***Avepp%***	**Proportions per class%**	***π_j%***	***BIC***	***△BIC***	***OCC***	***E_j***
2Groups(33)	93.8-88.3	3128(69.2)-1394(30.8)	68.5-31.5	-19396		7.0-16.5	0.735
2Groups(32)	93.8-88.3	3128(69.2)-1394(30.8)	68.5-31.5	-19391	5	7.0-16.5	0.735
2Groups(22)	93.8-88.3	3128(69.2)-1394(30.8)	68.5-31.5	-19386	5	7.0-16.5	0.735
2Groups(21)	93.8-88.4	3155(69.8)-1367(30.2)	69.0-31.0	-19398	-12	6.8-16.9	0.737
2Groups(11)	93.8-88.1	3143(69.5)-1379(30.5)	68.8-31.2	-19444	-46	6.9-16.3	0.733
3Groups(333)	89.0-85.1-87.3	2172(48.0)-1978(43.7)-372(8.2)	47.7-43.5-8.8	-18937	508	8.9-7.4-71.9	0.731
3Groups(323)	89.0-85.1-87.3	2172(48.0)-1978(43.7)-372(8.2)	47.7-43.5-8.8	-18932	5	8.9-7.4-71.9	0.731
3Groups(322)	89.0-85.1-87.3	2172(48.0)-1978(43.7)-372(8.2)	47.7-43.5-8.8	-18927	5	8.9-7.4-71.9	0.731

**Model**	***Avepp%***	**Proportions per class%**	***π_j%***	***BIC***	***⊿BIC***	***OCC***	***E_j***
3Groups(313)	89.4-84.7-87.9	2201(48.7)-1964(43.4)-357(7.9)	48.6-42.9-8.6	-18952	-24	9.0-7.4-77.1	0.731
3Groups(312)	89.4-84.7-87.9	2201(48.7)-1964(43.4)-357(7.9)	48.6-42.9-8.6	-18947	5	9.0-7.4-77.1	0.731
3Groups(311)	89.4-84.9-87.6	2213(48.9)-1956(43.3)-353(7.8)	48.8-42.8-8.4	-18947	0	8.9-7.5-77.1	0.733
3Groups(222)	89.0-85.1-87.3	2172(48.0)-1978(43.7)-372(8.2)	47.7-43.5-8.8	-18923	24	8.9-7.4-71.9	0.731
**3Groups(221)**	**89.1-85.2-87.2**	**2183(48.3)-1971(43.6)-368(8.1)**	**47.9-43.5-8.6**	**-18922**	**1**	**8.8-7.5-72.0**	0.732
3Groups(212)	89.4-84.7-87.9	2201(48.7)-1964(43.4)-357(7.9)	48.6-42.9-8.6	-18942	-21	9.0-7.4-77.1	0.731
3Groups(211)	89.4-84.9-87.6	2213(48.9)-1956(43.3)-353(7.8)	48.8-42.8-8.4	-18942	0	8.9-7.5-77.1	0.733
3Groups(111)	88.8-85.0-87.2	2204(48.7)-1958(43.3)-360(8.0)	48.2-43.2-8.5	-18993	-51	8.5-7.4-73.5	0.728
4Groups(3331)	82.6-78.5-83.3-88.5	1261(27.9)-2171(48.0)-971(21.5)-119(2.6)	29.0-45.6-22.5-2.9	-18810	183	11.6-4.4-17.2-253.2	0.686
4Groups(3231)	86.8-82.0-84.6-92.5	1782(39.4)-2063(45.6)-629(13.9)-48(1.1)	39.6-44.5-14.7-1.2	-18787	23	10.0-5.7-32.1-1021.4	0.741
4Groups(3221)	86.8-82.0-84.6-92.5	1783(39.4)-2062(45.6)-629(13.9)-48(1.1)	39.6-44.5-14.7-1.2	-18782	5	10.0-5.7-32.1-1021.3	0.741
4Groups(3211)	87.0-82.4-84.4-90.9	1817(40.2)-2053(45.4)-604(13.4)-48(1.1)	40.3-44.5-14.0-1.1	-18785	-3	9.9-5.8-33.2-870.3	0.745

Model	*Avepp%*	Proportions per class%	π_j%	*BIC*	*△BIC*	*OCC*	E_j
4Groups(3111)	98.6-90.3-86.5-88.9	89(2.0)-2167(47.9)-1876(41.5)-390(8.6)	2.3-47.4-41.4-9.0	-18435	349	2950.2-10.3-9.1-80.8	0.807
4Groups(1211)	86.6-82.0-84.3-90.9	1748(38.7)-2093(46.3)-631(14.0)-50(1.1)	39.1-45.1-14.6-1.2	-18823	-388	10.1-5.6-31.5-816.9	0.738
4Groups(1111)	86.7-81.9-84.4-91.3	1821(40.3)-2045(45.2)-608(13.4)-48(1.1)	40.3-44.3-14.2-1.2	-18855	-32	9.6-5.7-32.6-891.7	0.738

Avepp, average posterior probability; πj, observed group proportion; BIC, Bayesian Information Criterion; ΔBIC, change in BIC; OCC, odds of correct classification; Ej, classification error index. Model fit was evaluated using Bayesian Information Criterion (BIC), average posterior probability of assignment (Avepp, odds of correct classification (OCC), and class proportions. The optimal trajectory model was selected based on lower BIC values, acceptable classification probabilities, and balanced group sizes. BRI, body roundness index.

Table S2 Parameter estimates of group-based trajectory models for BRI

Group	Parmeter	Estimate	Standard Error	*t*	*P*
2_33					
Group1	Intercept	2.90411	269.24647	0.011	0.991
	Linear	0.09828	493.61841	0.000	1.000
	Quadratic	0.27373	269.24650	0.001	0.999
	Cubic	-0.08765	44.87442	-0.002	0.998
Group2	Intercept	4.72205	1585.4671	0.003	0.998
	Linear	0.10508	2906.6898	0.000	1.000
	Quadratic	0.28846	1585.4671	0.000	1.000
	Cubic	-0.08523	264.24453	-0.000	1.000
2_32					

**Group**	**Parmeter**	**Estimate**	**Standard Error**	***t***	***P***
Group1	Intercept	2.90411	299.60227	0.010	0.992
	Linear	0.09829	549.27081	0.000	1.000
	Quadratic	0.27373	299.60226	0.001	0.999
	Cubic	-0.08765	49.93371	-0.002	0.999
Group2	Intercept	4.21064	0.13510	31.167	**<0.001**
	Linear	1.04266	0.15487	6.733	**<0.001**
	Quadratic	-0.22294	0.03857	-5.780	**<0.001**
2_22					
Group1	Intercept	2.37820	0.08853	26.863	**<0.001**
	Linear	1.06245	0.10193	10.423	**<0.001**
	Quadratic	-0.25218	0.02541	-9.925	**<0.001**
Group2	Intercept	4.21064	0.13509	31.168	**<0.001**
	Linear	1.04266	0.15486	6.733	**<0.001**
	Quadratic	-0.22294	0.03857	-5.780	**<0.001**
2_21					
Group1	Intercept	2.36884	0.08858	26.743	**<0.001**
	Linear	1.08120	0.10193	10.607	**<0.001**
	Quadratic	-0.25658	0.02541	-10.098	**<0.001**
Group2	Intercept	4.94732	0.05058	97.804	**<0.001**
	Linear	0.15581	0.02108	7.393	**<0.001**
2_11					
Group1	Intercept	3.20076	0.03139	101.953	**<0.001**
	Linear	0.06076	0.01387	4.380	**<0.001**

Group	Parmeter	Estimate	Standard Error	*t*	*P*
Group2	Intercept	4.93988	0.05071	97.423	**<0.001**
	Linear	0.15687	0.02123	7.390	**<0.001**
3_333					
Group1	Intercept	2.57643	437.95859	0.006	0.995
	Linear	0.15841	802.92408	0.000	1.000
	Quadratic	0.25640	437.95899	0.001	1.000
	Cubic	-0.08937	72.99310	-0.001	0.999
Group2	Intercept	3.93956	710.58971	0.006	0.996
	Linear	0.15117	1302.7478	0.000	1.000
	Quadratic	0.24693	710.58971	0.000	1.000
	Cubic	-0.07682	118.43162	-0.001	0.999
Group3	Intercept	5.66746	929.91109	0.006	0.995
	Linear	0.29250	1704.8370	0.000	1.000
	Quadratic	0.14669	929.91110	0.000	1.000
	Cubic	-0.05474	154.98518	-0.000	1.000
3_323					
Group1	Intercept	2.57643	160.01212	0.016	0.987
	Linear	0.15841	293.35557	0.001	1.000
	Quadratic	0.25640	160.01213	0.002	0.999
	Cubic	-0.08937	26.66869	-0.003	0.997
Group2	Intercept	3.47863	0.11033	31.530	**<0.001**
	Linear	0.99621	0.12244	8.136	**<0.001**
	Quadratic	-0.21400	0.03054	-7.007	**<0.001**

**Group**	**Parmeter**	**Estimate**	**Standard Error**	***t***	***P***
Group3	Intercept	5.66746	1530.3998	0.004	0.997
	Linear	0.29250	2805.7331	0.000	1.000
	Quadratic	0.14669	1530.3998	0.000	1.000
	Cubic	-0.05474	255.06664	-0.000	1.000
3_322					
Group1	Intercept	2.57643	256.13740	0.010	0.992
	Linear	0.15841	469.58524	0.000	1.000
	Quadratic	0.25639	256.13740	0.001	0.999
	Cubic	-0.08937	42.68957	-0.002	0.998
Group2	Intercept	3.47864	0.11032	31.532	**<0.001**
	Linear	0.99621	0.12244	8.136	**<0.001**
	Quadratic	-0.21400	0.03054	-7.008	**<0.001**
Group3	Intercept	5.33901	0.23623	22.600	**<0.001**
	Linear	0.89467	0.27022	3.311	**<0.001**
	Quadratic	-0.18177	0.06724	-2.703	**0.006**
3_313					
Group1	Intercept	2.56992	257.32602	0.010	0.992
	Linear	0.16289	471.76436	0.000	1.000
	Quadratic	0.27036	257.32601	0.001	0.999
	Cubic	-0.09390	42.88767	-0.002	0.998
Group2	Intercept	4.18786	0.04795	87.344	**<0.001**
	Linear	0.14658	0.01669	8.783	**<0.001**
Group3	Intercept	5.64722	1561.5768	0.004	0.997

Group	Parmeter	Estimate	Standard Error	*t*	*P*
	Linear	0.29645	2862.8908	0.000	1.000
	Quadratic	0.17282	1561.5768	0.000	1.000
	Cubic	-0.06289	260.26280	-0.000	1.000
3_312					
Group1	Intercept	2.56992	168.79312	0.015	0.988
	Linear	0.16289	309.45405	0.001	1.000
	Quadratic	0.27036	168.79312	0.002	0.999
	Cubic	-0.09390	28.13219	-0.003	0.997
Group2	Intercept	4.18786	0.04794	87.347	**<0.001**
	Linear	0.14659	0.01669	8.784	**<0.001**
Group3	Intercept	5.26989	0.23824	22.120	**<0.001**
	Linear	0.98822	0.27209	3.632	**<0.001**
	Quadratic	-0.20451	0.06778	-3.017	**0.002**
3_311					
Group1	Intercept	2.57235	138.20794	0.019	0.985
	Linear	0.16375	253.38124	0.001	0.999
	Quadratic	0.26954	138.20795	0.002	0.998
	Cubic	-0.09369	23.03466	-0.004	0.997
Group2	Intercept	4.19443	0.04768	87.977	**<0.001**
	Linear	0.14702	0.01666	8.822	**<0.001**
Group3	Intercept	5.95041	0.08976	66.295	**<0.001**
	Linear	0.17529	0.03710	4.724	**<0.001**
3_222					

**Group**	**Parmeter**	**Estimate**	**Standard Error**	***t***	***P***
Group1	Intercept	2.04023	0.09834	20.747	**<0.001**
	Linear	1.14144	0.11198	10.194	**<0.001**
	Quadratic	-0.27980	0.02797	-10.004	**<0.001**
Group2	Intercept	3.47862	0.11032	31.533	**<0.001**
	Linear	0.99622	0.12243	8.137	**<0.001**
	Quadratic	-0.21400	0.03054	-7.008	**<0.001**
Group3	Intercept	5.33899	0.23623	22.601	**<0.001**
	Linear	0.89471	0.27021	3.311	**<0.001**
	Quadratic	-0.18178	0.06724	-2.703	**0.006**
**3_221**					
**Group1**	**Intercept**	**2.04416**	**0.09817**	**20.823**	<0.001
	**Linear**	**1.13946**	**0.11179**	**10.193**	<0.001
	**Quadratic**	**-0.27923**	**0.02792**	**-10.000**	<0.001
**Group2**	**Intercept**	**3.47117**	**0.11032**	**31.463**	<0.001
	**Linear**	**1.01241**	**0.12253**	**8.263**	<0.001
	**Quadratic**	**-0.21793**	**0.03056**	**-7.132**	<0.001
**Group3**	**Intercept**	**5.94330**	**0.08803**	**67.517**	<0.001
	**Linear**	**0.17107**	**0.03630**	**4.713**	<0.001
3_212					
Group1	Intercept	2.00650	0.09777	20.522	**<0.001**
	Linear	1.19583	0.11118	10.756	**<0.001**
	Quadratic	-0.29306	0.02778	-10.548	**<0.001**
Group2	Intercept	4.18786	0.04794	87.351	**<0.001**

**Group**	**Parmeter**	**Estimate**	**Standard Error**	***t***	***P***
	Linear	0.14658	0.01669	8.784	**<0.001**
Group3	Intercept	5.26990	0.23823	22.121	**<0.001**
	Linear	0.98821	0.27208	3.632	**<0.001**
	Quadratic	-0.20451	0.06778	-3.017	**0.002**
3_211					
Group1	Intercept	2.01022	0.09760	20.596	**<0.001**
	Linear	1.19432	0.11100	10.760	**<0.001**
	Quadratic	-0.29259	0.02774	-10.548	**<0.001**
Group2	Intercept	4.19443	0.04767	87.980	**<0.001**
	Linear	0.14701	0.01666	8.823	**<0.001**
Group3	Intercept	5.95040	0.08975	66.298	**<0.001**
	Linear	0.17530	0.03710	4.725	**<0.001**
3_111					
Group1	Intercept	2.95229	0.03585	82.353	**<0.001**
	Linear	0.03161	0.01564	2.022	**0.043**
Group2	Intercept	4.18304	0.04840	86.425	**<0.001**
	Linear	0.14611	0.01682	8.687	**<0.001**
Group3	Intercept	5.94052	0.08997	66.031	**<0.001**
	Linear	0.17496	0.03708	4.718	**<0.001**
4_3331					
Group1	Intercept	2.31214	179.25486	0.013	0.990
	Linear	0.17506	328.63405	0.001	1.000
	Quadratic	0.27630	179.25496	0.002	0.999

**Group**	**Parmeter**	**Estimate**	**Standard Error**	***t***	***P***
	Cubic	-0.10101	29.87583	-0.003	0.997
Group2	Intercept	3.36470	449.94236	0.007	0.994
	Linear	0.13398	824.89446	0.000	1.000
	Quadratic	0.24454	449.94243	0.001	1.000
	Cubic	-0.07564	74.99041	-0.001	0.999
Group3	Intercept	4.63740	640.57677	0.007	0.994
	Linear	0.21851	1174.3905	0.000	1.000
	Quadratic	0.21913	640.57663	0.000	1.000
	Cubic	-0.07306	106.76277	-0.001	0.999
Group4	Intercept	6.65780	0.52855	12.596	**<0.001**
	Linear	0.20286	0.06073	3.340	**<0.001**
4_3231					
Group1	Intercept	2.43383	281.94870	0.009	0.993
	Linear	0.21613	516.90596	0.000	1.000
	Quadratic	0.23210	281.94874	0.001	0.999
	Cubic	-0.08779	46.99145	-0.002	0.999
Group2	Intercept	3.19934	0.10937	29.251	**<0.001**
	Linear	1.00006	0.11890	8.411	**<0.001**
	Quadratic	-0.21539	0.02963	-7.269	**<0.001**
Group3	Intercept	5.01254	1031.4823	0.005	0.996
	Linear	0.36689	1891.0508	0.000	1.000
	Quadratic	0.11933	1031.4822	0.000	1.000
	Cubic	-0.05321	171.91371	-0.000	1.000

**Group**	**Parmeter**	**Estimate**	**Standard Error**	***t***	***P***
Group4	Intercept	7.34966	0.23272	31.581	**<0.001**
	Linear	0.21883	0.09883	2.214	**0.026**
4_3221					
Group1	Intercept	2.43384	206.17394	0.012	0.991
	Linear	0.21613	377.98553	0.001	1.000
	Quadratic	0.23210	206.17393	0.001	0.999
	Cubic	-0.08779	34.36232	-0.003	0.998
Group2	Intercept	3.19936	0.10937	29.253	**<0.001**
	Linear	1.00004	0.11889	8.411	**<0.001**
	Quadratic	-0.21539	0.02963	-7.269	**<0.001**
Group3	Intercept	4.69328	0.19014	24.683	**<0.001**
	Linear	0.95221	0.20681	4.604	**<0.001**
	Quadratic	-0.19993	0.05145	-3.886	**<0.001**
Group4	Intercept	7.34967	0.23271	31.583	**<0.001**
	Linear	0.21885	0.09883	2.214	**0.026**
4_3211					
Group1	Intercept	2.44232	231.74038	0.011	0.992
	Linear	0.21890	424.85738	0.001	1.000
	Quadratic	0.22884	231.74039	0.001	0.999
	Cubic	-0.08687	38.62340	-0.002	0.998
Group2	Intercept	3.19296	0.10872	29.367	**<0.001**
	Linear	1.03573	0.11847	8.743	**<0.001**
	Quadratic	-0.22414	0.02954	-7.589	**<0.001**

**Group**	**Parmeter**	**Estimate**	**Standard Error**	***t***	***P***
Group3	Intercept	5.37853	0.08105	66.358	**<0.001**
	Linear	0.15638	0.02820	5.546	**<0.001**
Group4	Intercept	7.37068	0.23572	31.269	**<0.001**
	Linear	0.22765	0.10070	2.261	**0.023**
4_3111					
Group1	Intercept	2.37492	779.21023	0.003	0.998
	Linear	0.84358	1428.5522	0.001	1.000
	Quadratic	1.34667	779.21028	0.002	0.999
	Cubic	-0.65577	129.86838	-0.005	0.996
Group2	Intercept	2.78134	0.03328	83.578	**<0.001**
	Linear	0.13542	0.01392	9.725	**<0.001**
Group3	Intercept	4.18908	0.04299	97.445	**<0.001**
	Linear	0.15293	0.01526	10.022	**<0.001**
Group4	Intercept	5.91919	0.07777	76.112	**<0.001**
	Linear	0.17412	0.03245	5.366	**<0.001**
4_1211					
Group1	Intercept	2.85681	0.03969	71.976	**<0.001**
	Linear	0.01081	0.01753	0.617	0.538
Group2	Intercept	3.07455	0.10928	28.135	**<0.001**
	Linear	1.14116	0.11749	9.712	**<0.001**
	Quadratic	-0.25112	0.02929	-8.573	**<0.001**
Group3	Intercept	5.34404	0.08351	63.994	**<0.001**
	Linear	0.15598	0.02772	5.627	**<0.001**

Group	Parmeter	Estimate	Standard Error	*t*	*P*
Group4	Intercept	7.33213	0.23667	30.980	**<0.001**
	Linear	0.22274	0.09922	2.245	**0.024**
4_1111					
Group1	Intercept	2.86827	0.03907	73.421	**<0.001**
	Linear	0.01416	0.01717	0.825	0.410
Group2	Intercept	3.91268	0.05203	75.194	**<0.001**
	Linear	0.14437	0.01657	8.712	**<0.001**
Group3	Intercept	5.35516	0.08365	64.021	**<0.001**
	Linear	0.15930	0.02824	5.641	**<0.001**
Group4	Intercept	7.35696	0.23851	30.846	**<0.001**
	Linear	0.22563	0.10100	2.234	**0.025**

Trajectory models were estimated using polynomial functions (linear, quadratic, and cubic terms). Parameter estimates, standard errors, t statistics, and P values are reported for each trajectory group. BRI, body roundness index.

Table S3 Overall performance of individual and stacking ensemble machine learning models for hypertension prediction.

Number	Model	AUC	Accuracy	F1score	NPV	PPV	Sensitivity	Specificity
1	SVM	0.63-	0.63	0.00	0.63	0.00	0.00	1.00
2	RF	0.73-	0.70	0.45	0.70	0.70	0.33	0.92
3	BDT	0.84-	0.83	0.72	0.81	0.89	0.61	0.96

4	DT	0.68-	0.71	0.54	0.73	0.64	0.47	0.85
5	LR	0.62-	0.64	0.11	0.64	0.57	0.06	0.97
6	SVM-RF	0.73-	0.70	0.46	0.70	0.70	0.34	0.92
7	SVM-BDT	0.84-	0.83	0.73	0.81	0.88	0.62	0.95
8	SVM-DT	0.71-	0.72	0.55	0.73	0.67	0.47	0.86
9	SVM-LR	0.62-	0.63	0.13	0.64	0.52	0.08	0.96
10	RF-BDT	0.83-	0.82	0.72	0.81	0.87	0.62	0.94
11	RF-DT	0.74-	0.72	0.52	0.72	0.71	0.41	0.90
12	RF-LR	0.72-	0.70	0.46	0.70	0.70	0.34	0.91
13	BDT-DT	0.84-	0.83	0.73	0.81	0.87	0.62	0.95
14	BDT-LR	0.84-	0.83	0.73	0.81	0.88	0.62	0.95
15	DT-LR	0.71-	0.72	0.54	0.73	0.67	0.46	0.87
16	SVM-RF-BDT	0.83-	0.82	0.72	0.81	0.87	0.62	0.94

17	SVM-RF-DT	0.74-	0.72	0.52	0.72	0.70	0.41	0.90
18	SVM-RF-LR	0.72-	0.70	0.46	0.70	0.70	0.34	0.91
19	SVM-BDT-DT	0.84-	0.83	0.73	0.81	0.87	0.62	0.95
20	SVM-BDT-LR	0.84-	0.83	0.73	0.81	0.88	0.62	0.95
21	SVM-DT-LR	0.71-	0.72	0.55	0.73	0.67	0.46	0.87
22	RF-BDT-DT	0.83-	0.82	0.72	0.81	0.87	0.62	0.95
23	RF-BDT-LR	0.83-	0.82	0.72	0.81	0.87	0.62	0.95
24	RF-DT-LR	0.74-	0.72	0.51	0.72	0.71	0.40	0.90
25	BDT-DT-LR	0.84-	0.83	0.73	0.81	0.87	0.62	0.95
26	SVM-RF-BDT-DT	0.83-	0.82	0.72	0.81	0.87	0.62	0.95
27	SVM-RF-BDT-LR	0.83-	0.83	0.72	0.81	0.87	0.62	0.95
28	SVM-RF-DT-LR	0.74-	0.72	0.51	0.72	0.71	0.40	0.90
29	SVM-BDT-DT-LR	0.84-	0.83	0.73	0.81	0.87	0.62	0.95

30	RF-BDT-DT-LR	0.83-	0.83	0.72	0.81	0.87	0.62	0.95
31	SVM-RF-BDT-DT-LR	0.83-	0.83	0.72	0.81	0.87	0.62	0.95

Model performance was evaluated using area under the receiver operating characteristic curve (AUC), accuracy, F1 score, negative predictive value (NPV), positive predictive value (PPV), sensitivity, and specificity. SVM, support vector machine; RF, random forest; BDT, boosted decision tree; DT, decision tree; LR, logistic regression.

Table S4 Comprehensive rank among different ML models.

**Model**	**AUC Rank**	**Accuracy Rank**	**F1score Rank**	**NPV Rank**	**PPV Rank**	**Sensitivity Rank**	**Specificity Rank**	**Comprehensive Rank**
SVM BDT DT LR	7.00	3.50	1.50	1.50	5.50	1.50	10.50	4.43
BDT DT LR	8.00	3.50	1.50	1.50	5.50	1.50	10.50	4.57
SVM BDT	2.00	3.50	6.00	6.00	3.00	7.00	6.00	4.79
BDT LR	3.00	3.50	6.00	6.00	3.00	7.00	6.00	4.93
SVM BDT LR	4.00	3.50	6.00	6.00	3.00	7.00	6.00	5.07
SVM BDT DT	5.50	7.50	3.50	3.50	7.50	3.50	10.50	5.93

BDT DT	5.50	7.50	3.50	3.50	7.50	3.50	10.50	5.93
BDT	1.00	3.50	11.00	16.00	1.00	16.00	4.00	7.50
SVM RF BDT LR	13.50	10.00	10.00	10.00	9.00	11.00	10.50	10.57
SVM RF BDT DT LR	15.00	10.00	8.50	8.50	11.50	7.00	15.50	10.86
RF BDT DT LR	16.00	10.00	8.50	8.50	11.50	7.00	15.50	11.00
RF BDT LR	13.50	12.00	12.00	13.00	10.00	14.00	10.50	12.14
SVM RF BDT DT	11.50	13.50	13.50	14.50	13.50	14.00	15.50	13.71
RF BDT DT	11.50	13.50	13.50	14.50	13.50	14.00	15.50	13.71
SVM RF BDT	9.50	15.50	15.50	11.50	15.50	11.00	18.50	13.86
RF BDT	9.50	15.50	15.50	11.50	15.50	11.00	18.50	13.86
RF DT	17.00	17.50	21.00	21.00	18.00	21.50	25.50	20.21
RF DT LR	20.00	17.50	23.00	23.00	17.00	23.50	24.00	21.14
SVM RF DT	18.00	19.50	22.00	22.00	20.00	21.50	27.00	21.43

SVM RF DT LR	19.00	19.50	24.00	24.00	19.00	23.50	25.50	22.07
SVM DT LR	26.00	21.00	18.00	18.00	25.00	19.50	28.00	22.21
SVM DT	27.00	22.50	17.00	17.00	27.00	18.00	30.00	22.64
DT LR	25.00	22.50	19.00	19.00	26.00	19.50	29.00	22.86
DT	28.00	24.00	20.00	20.00	28.00	17.00	31.00	24.00
SVM RF	21.50	25.50	26.00	26.00	22.00	26.50	21.00	24.07
RF LR	23.00	25.50	25.00	25.00	23.00	25.00	22.50	24.14
RF	21.50	28.00	28.00	28.00	21.00	28.00	20.00	24.93
SVM RF LR	24.00	27.00	27.00	27.00	24.00	26.50	22.50	25.43
LR	30.00	29.00	30.00	30.00	29.00	30.00	2.00	25.71
SVM LR	31.00	30.00	29.00	29.00	30.00	29.00	3.00	25.86
SVM	29.00	31.00	31.00	31.00	31.00	31.00	1.00	26.43

Models were ranked according to AUC, accuracy, F1 score, NPV, PPV, sensitivity, and specificity. A lower comprehensive rank indicates better overall predictive performance. AUC, area under the receiver operating characteristic curve; PPV, positive predictive value; NPV, negative predictive value. SVM, support vector machine; RF, random forest; BDT, boosted decision tree; DT, decision tree; LR, logistic regression.

Table S5 Association between BRI trajectory and hypertension incidence in a population excluding participants with any missing values

Variable	crude model		Model 1		Model 2		Model 3	
	OR (95%CI)	***P***	***OR (95%CI)***	***P***	***OR (95%CI)***	***P***	***OR (95%CI)***	***P***
Trajectory								
1	ref		ref		ref		ref	
2	1.56(1.26,1.93)	<0.0001	1.63(1.31,2.04)	<0.0001	1.57(1.25,1.97)	<0.001	1.56(1.24,1.96)	<0.001
3	2.23(1.60,3.12)	<0.0001	2.3(1.62,3.26)	<0.0001	2.18(1.52,3.11)	<0.0001	2.10(1.46,3.01)	<0.0001
p for trend		<0.0001		<0.0001		<0.0001		<0.0001

Model 1 was adjusted for age, sex, marital status, residence, and education; Model 2 was additionally adjusted for CRP, triglycerides, remnant cholesterol, smoking, and alcohol consumption; Model 3 was further adjusted for dyslipidemia, heart disease, stroke, kidney disease, and lung disease. OR (95%CI), odds ratios and 95% confidence intervals; BRI, body roundness index.

Table S6 Association between baseline BRI and hypertension incidence

Variable	crude model		Model 1		Model 2		Model 3	
	OR (95%CI)	P	OR (95%CI)	P	OR (95%CI)	P	OR (95%CI)	P
BRI (per 1 unit)	1.19(1.12,1.27)	<0.0001	1.2(1.12,1.28)	<0.0001	1.18(1.10,1.26)	<0.0001	1.18(1.10,1.26)	<0.0001
BRI (per 1-SD)	1.26(1.16,1.37)	<0.0001	1.27(1.17,1.38)	<0.0001	1.24(1.14,1.36)	<0.0001	1.24(1.14,1.35)	<0.0001
BRI (tertiles)								
T1	ref		ref		ref		ref	
T2	1.27(1.03,1.57)	0.03	1.29(1.04,1.60)	0.02	1.26(1.02,1.56)	0.04	1.27(1.02,1.57)	0.03
T3	1.75(1.43,2.15)	<0.0001	1.79(1.45,2.22)	<0.0001	1.7(1.37,2.11)	<0.0001	1.69(1.36,2.10)	<0.0001
p for trend		<0.0001		<0.0001		<0.0001		<0.0001

Baseline BRI was analyzed as a continuous variable (per 1-unit increase and per 1-SD increase) and as tertiles. Model 1 was adjusted for age, sex, marital status, residence, and education; Model 2 was additionally adjusted for c-reactive protein, remnant cholesterol, triglycerides, remnant cholesterol, smoking, and alcohol consumption; Model 3 was further adjusted for dyslipidemia, heart disease, stroke, kidney disease, and lung disease. BRI, body roundness index.

Table S7 Association between BRI trajectory and hypertension incidence in a population after removing all participants who used lipid-lowering drugs

**Variable**	**crude model**		**Model 1**		**Model 2**		**Model 3**	
	OR (95%CI)	***P***	***OR (95%CI)***	***P***	***OR (95%CI)***	***P***	***OR (95%CI)***	***P***
Trajectory								
1	ref		ref		ref		ref	
2	1.50(1.26,1.79)	<0.0001	1.57(1.31, 1.88)	<0.0001	1.50(1.25, 1.81)	<0.0001	1.50(1.25,1.81)	<0.0001
3	2.09(1.57,2.77)	<0.0001	2.17(1.61,1.93)	<0.0001	2.07(1.53,1.79)	<0.0001	2.05(1.51,1.77)	<0.0001
p for trend		<0.0001		<0.0001		<0.0001		<0.0001

Model 1 was adjusted for age, sex, marital status, residence, and education; Model 2 was additionally adjusted for c-reactive protein, remnant cholesterol, triglycerides, remnant cholesterol, smoking, and alcohol consumption; Model 3 was further adjusted for dyslipidemia, heart disease, stroke, kidney disease, and lung disease. OR (95%CI), odds ratios and 95% confidence intervals; BRI, body roundness index.

Table S8 Association between BRI trajectory and hypertension incidence in a population after removing all participants who has cancer

**Variable**	**crude model**		**Model 1**		**Model 2**		**Model 3**	
	OR (95%CI)	***P***	***OR (95%CI)***	***P***	***OR (95%CI)***	***P***	***OR (95%CI)***	***P***
Trajectory								
1	ref		ref		ref		ref	

Variable	crude model		Model 1		Model 2		Model 3	
2	1.56(1.26,1.93)	<0.0001	1.63(1.31,2.04)	<0.0001	1.57(1.25,1.97)	<0.001	1.56(1.24,1.96)	<0.001
3	2.23(1.60,3.12)	<0.0001	2.3(1.62,3.26)	<0.0001	2.18(1.52,3.11)	<0.0001	2.1(1.46,3.01)	<0.0001
p for trend		<0.0001		<0.0001		<0.0001		<0.0001

Model 1 was adjusted for age, sex, marital status, residence, and education; Model 2 was additionally adjusted for c-reactive protein, remnant cholesterol, triglycerides, remnant cholesterol, smoking, and alcohol consumption; Model 3 was further adjusted for dyslipidemia, heart disease, stroke, kidney disease, and lung disease. OR (95%CI), odds ratios and 95% confidence intervals; BRI, body roundness index.

Table S9 Weighted logistic regression confirmed a positive association between BRI trajectory and hypertension risk

**Group**	***OR 95%CI***	***p***
Traj 1	ref	
Traj 2	1.05(1.03, 1.07)	<0.001
Traj 3	1.12(1.05, 1.20)	<0.0001

Survey-weighted logistic regression models were applied to account for the complex sampling design. Odds ratios and 95% confidence intervals were estimated for each BRI trajectory group using the low-level inverted U-shaped trajectory as the reference. Traj, trajectory; OR (95%CI), odds ratios and 95% confidence intervals.

Table S10 Association between BRI trajectory and hypertension incidence after excluding potential metabolic and clinical intermediates

Variable	crude model		Model 1		Model 2		Model 3	
	OR (95%CI)	***P***	***OR (95%CI)***	***P***	***OR (95%CI)***	***P***	***OR (95%CI)***	***P***
Trajectory								
1	ref		ref		ref		ref	
2	1.51(1.27,1.80)	<0.0001	1.57(1.31,1.88)	<0.0001	1.56(1.30,1.87)	<0.0001	1.57(1.31,1.88)	<0.0001
3	2.13(1.61,2.80)	<0.0001	2.21(1.66,2.95)	<0.0001	2.20(1.64,2.93)	<0.0001	2.20(1.64,2.94)	<0.0001
p for trend		<0.0001		<0.0001		<0.0001		<0.0001

Crude model includes only the trajectory of BRI variable; Model 1 was adjusted for age, sex, marital status, residence, and education; Model 2 was additionally adjusted for smoking, and alcohol consumption; Model 3 was further adjusted for stroke, kidney disease, and lung disease. OR (95%CI), odds ratios and 95% confidence intervals; BRI, body roundness index.

Table S11 Cox proportional hazards regression models confirmed a positive association between BRI trajectory and hypertension risk

Variable	crude model		Model 1		Model 2		Model 3	
	HR (95%CI)	***P***	***HR (95%CI)***	***P***	***HR (95%CI)***	***P***	***HR (95%CI)***	***P***
Trajectory								
1	ref		ref		ref		ref	
2	1.47(1.26,1.72)	<0.0001	1.53(1.30,1.80)	<0.0001	1.47(1.24,1.73)	<0.0001	1.46(1.23,1.72)	<0.0001
3	1.98(1.55,2.53)	<0.0001	2.05(1.58,2.65)	<0.0001	1.96(1.51,2.54)	<0.0001	1.92(1.48,2.50)	<0.0001
p for trend		<0.0001		<0.0001		0.01		0.01

Crude model includes only the trajectory of BRI variable; Model 1 was adjusted for age, sex, marital status, residence, and education; Model 2 was additionally adjusted for c-reactive protein, triglycerides, remnant cholesterol, smoking, and alcohol consumption; Model 3 was further adjusted for dyslipidemia, heart disease, stroke, kidney disease, and lung disease. HR, Hazard Ratio; CI, confidence intervals; BRI, body roundness index.

Figure S1

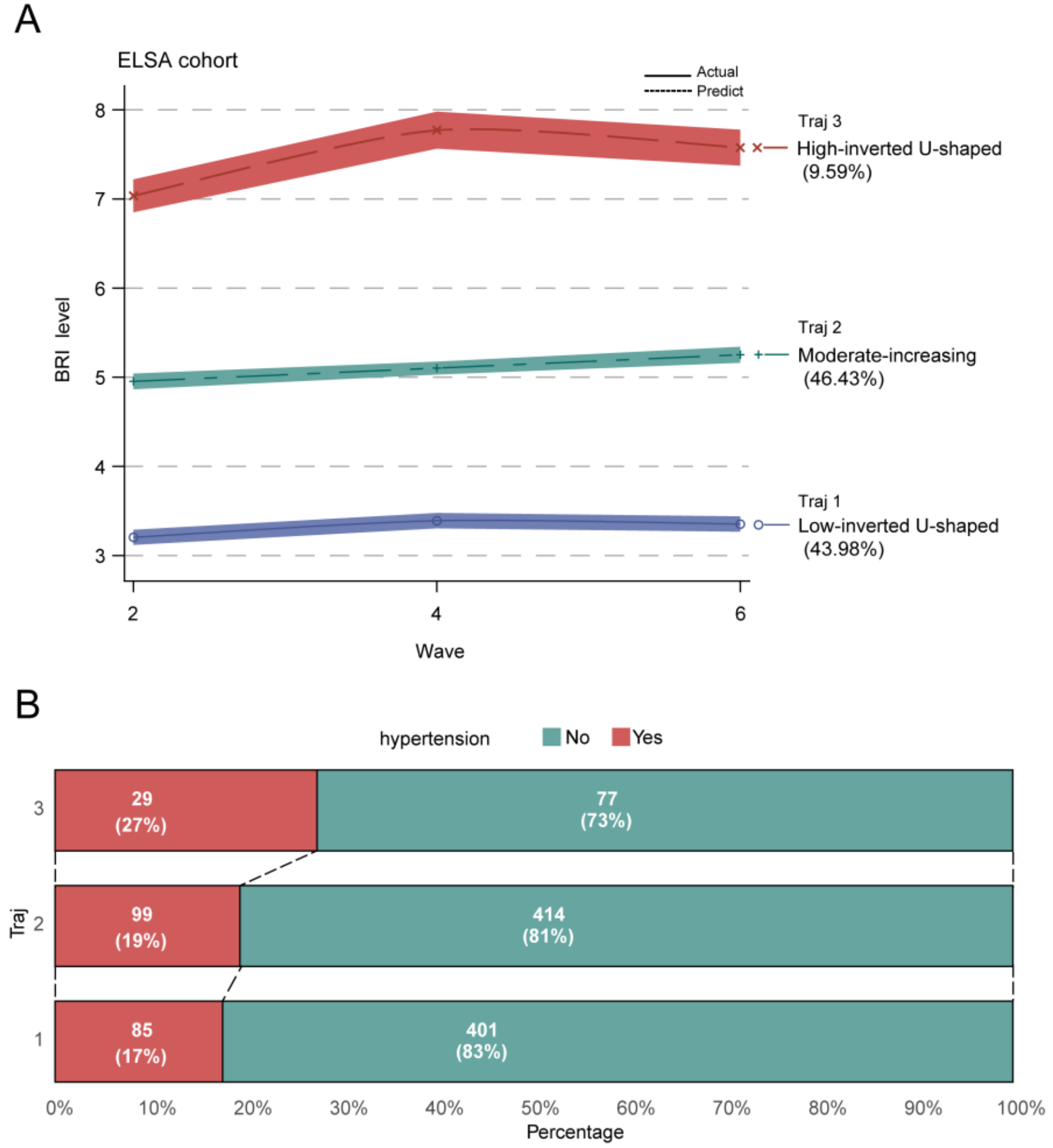
A
ELSA cohort
Actual
Predict
BRI level
8
7
6
5
4
3
Traj 3
High-inverted U-shaped
(9.59%)
Traj 2
Moderate-increasing
(46.43%)
Traj 1
Low-inverted U-shaped
(43.98%)
2
4
6
Wave
B
hypertension
No
Yes
Traj
3
2
1
29
(27%)
77
(73%)
99
(19%)
414
(81%)
85
(17%)
401
(83%)
0%
10%
20%
30%
40%
50%
60%
70%
80%
90%
100%
Percentage

Figure S1. Longitudinal trajectories of body roundness index and incidence of hypertension in the ELSA cohort. (A) Group-based trajectory modeling identified three distinct and stable BRI trajectories, classified as low-inverted U-shaped, moderate-increasing, and high-inverted U-shaped patterns. (B) The cumulative incidence of hypertension increased progressively across the three BRI trajectory groups, with the highest incidence observed in the high-inverted U-shaped trajectory group.

Figure S2

Variable	OR (95% CI)	P value	OR (95% CI)	P value	p for trend	p for interaction
**age**						0.30
45~60y	1.33(1.04,1.70)	0.02	2.00(1.34,2.94)	<0.001	<0.001	
>=60y	1.75(1.37,2.24)	<0.0001	2.26(1.52,3.32)	<0.0001	<0.0001	
**sex**						0.62
male	1.67(1.30,2.16)	<0.0001	2.01(1.02,3.69)	0.03	<0.0001	
female	1.43(1.11,1.85)	0.01	2.16(1.54,3.02)	<0.0001	<0.0001	
**education**						0.69
Middle school and below	1.50(1.25,1.80)	<0.0001	2.16(1.62,2.87)	<0.0001	<0.0001	
High school or above	1.65(0.94,2.95)	0.08	1.47(0.41,4.21)	0.51	0.13	
**drink**						0.91
no	1.50(1.21,1.88)	<0.001	2.09(1.51,2.88)	<0.0001	<0.0001	
yes	1.56(1.17,2.09)	0.003	2.42(1.31,4.26)	0.003	<0.001	
**smoke**						0.84
Never	1.42(1.13,1.80)	0.003	2.11(1.52,2.92)	<0.0001	<0.0001	
Current smoker	1.54(1.09,2.14)	0.01	2.17(0.90,4.68)	0.06	0.004	
Fomer smoker	1.89(1.19,3.04)	0.01	1.82(0.50,5.35)	0.31	0.01	
**dyslipidemia**						0.36
no	1.39(1.14,1.71)	0.001	2.19(1.56,3.03)	<0.0001	<0.0001	
yes	1.77(1.23,2.59)	0.003	1.97(1.16,3.31)	0.01	0.002	
**lung disease**						0.75
yes	1.76(1.13,2.76)	0.01	2.56(1.21,5.19)	0.01	0.002	
no	1.48(1.23,1.79)	<0.0001	2.08(1.53,2.79)	<0.0001	<0.0001	
**kidney disease**						0.53
no	1.49(1.24,1.79)	<0.0001	2.20(1.64,2.93)	<0.0001	<0.0001	
yes	1.73(1.00,3.02)	0.05	1.47(0.51,3.70)	0.43	0.1	
**heart disease**						0.25
no	1.43(1.19,1.72)	<0.001	2.15(1.59,2.89)	<0.0001	<0.0001	
yes	2.06(1.26,3.46)	0.005	1.88(0.85,3.95)	0.11	0.02	

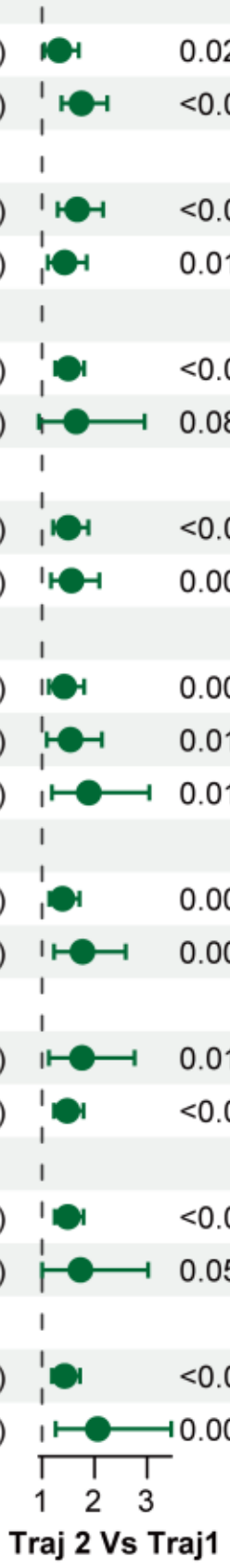

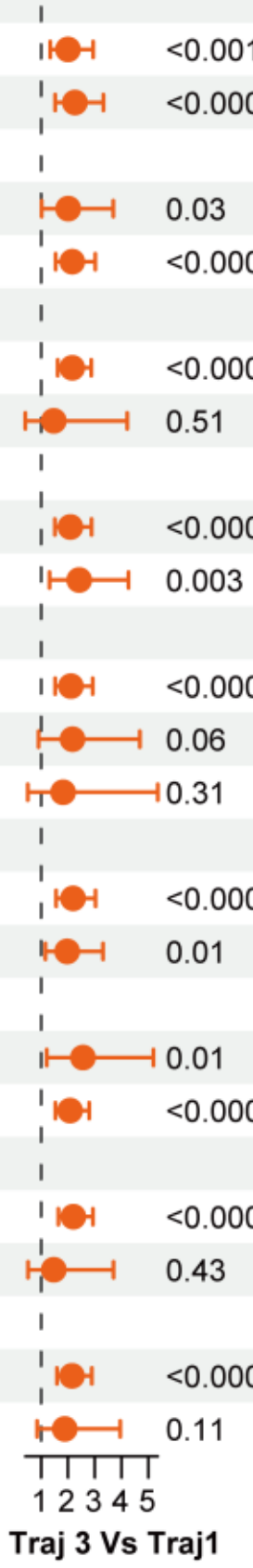

Figure S2. Subgroup analyses of the association between BRI trajectories and incident hypertension in the CHARLS cohort.

Figure S3

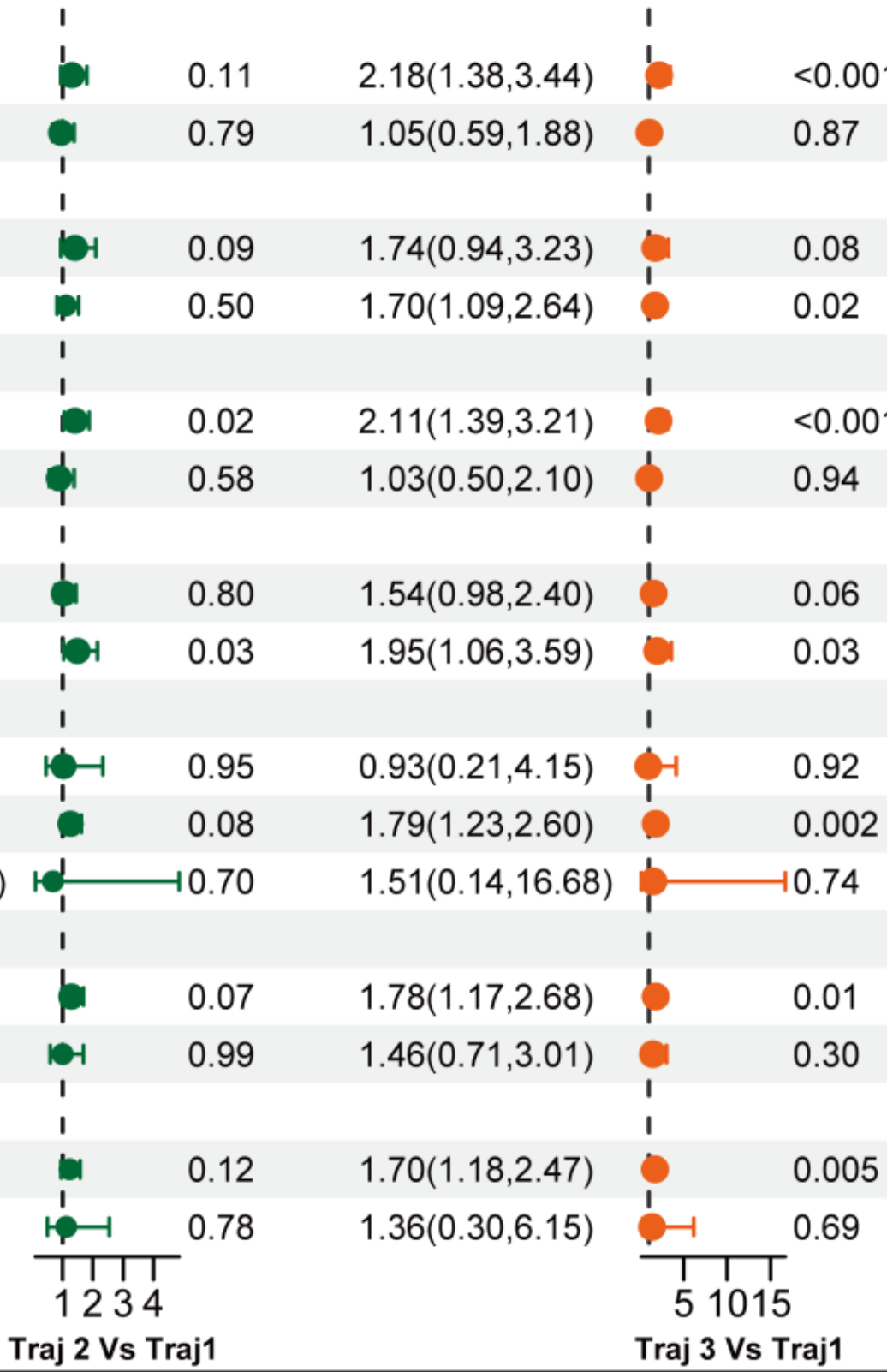

Variable	OR (95% CI)	P value	OR (95% CI)	P value	p for trend	p for interaction
**age**						0.13
60-70y	1.31(0.94,1.80)	0.11	2.18(1.38,3.44)	<0.001	0.001	
>=70y	0.95(0.66,1.38)	0.79	1.05(0.59,1.88)	0.87	1	
**sex**						0.63
male	1.41(0.94,2.10)	0.09	1.74(0.94,3.23)	0.08	0.04	
female	1.11(0.81,1.53)	0.50	1.70(1.09,2.64)	0.02	0.04	
**marital status**						0.11
Married	1.41(1.05,1.88)	0.02	2.11(1.39,3.21)	<0.001	<0.001	
Other	0.88(0.56,1.38)	0.58	1.03(0.50,2.10)	0.94	0.82	
**education**						0.36
No	1.04(0.75,1.44)	0.80	1.54(0.98,2.40)	0.06	0.12	
Yes	1.49(1.03,2.15)	0.03	1.95(1.06,3.59)	0.03	0.01	
**smoke**						0.88
Current	1.03(0.45,2.33)	0.95	0.93(0.21,4.15)	0.92	0.98	
Never	1.26(0.97,1.62)	0.08	1.79(1.23,2.60)	0.002	0.003	
Former	0.68(0.10, 4.85)	0.70	1.51(0.14,16.68)	0.74	0.89	
**drink**						0.70
Yes	1.29(0.98,1.69)	0.07	1.78(1.17,2.68)	0.01	0.005	
No	1.00(0.59,1.69)	0.99	1.46(0.71,3.01)	0.30	0.43	
**cancer**						0.95
No	1.23(0.95,1.58)	0.12	1.70(1.18,2.47)	0.005	0.01	
Yes	1.12(0.50,2.54)	0.78	1.36(0.30,6.15)	0.69	0.67	

Figure S3. Subgroup analyses of the association between BRI trajectories and incident hypertension in the ELSA cohort.

Figure S4

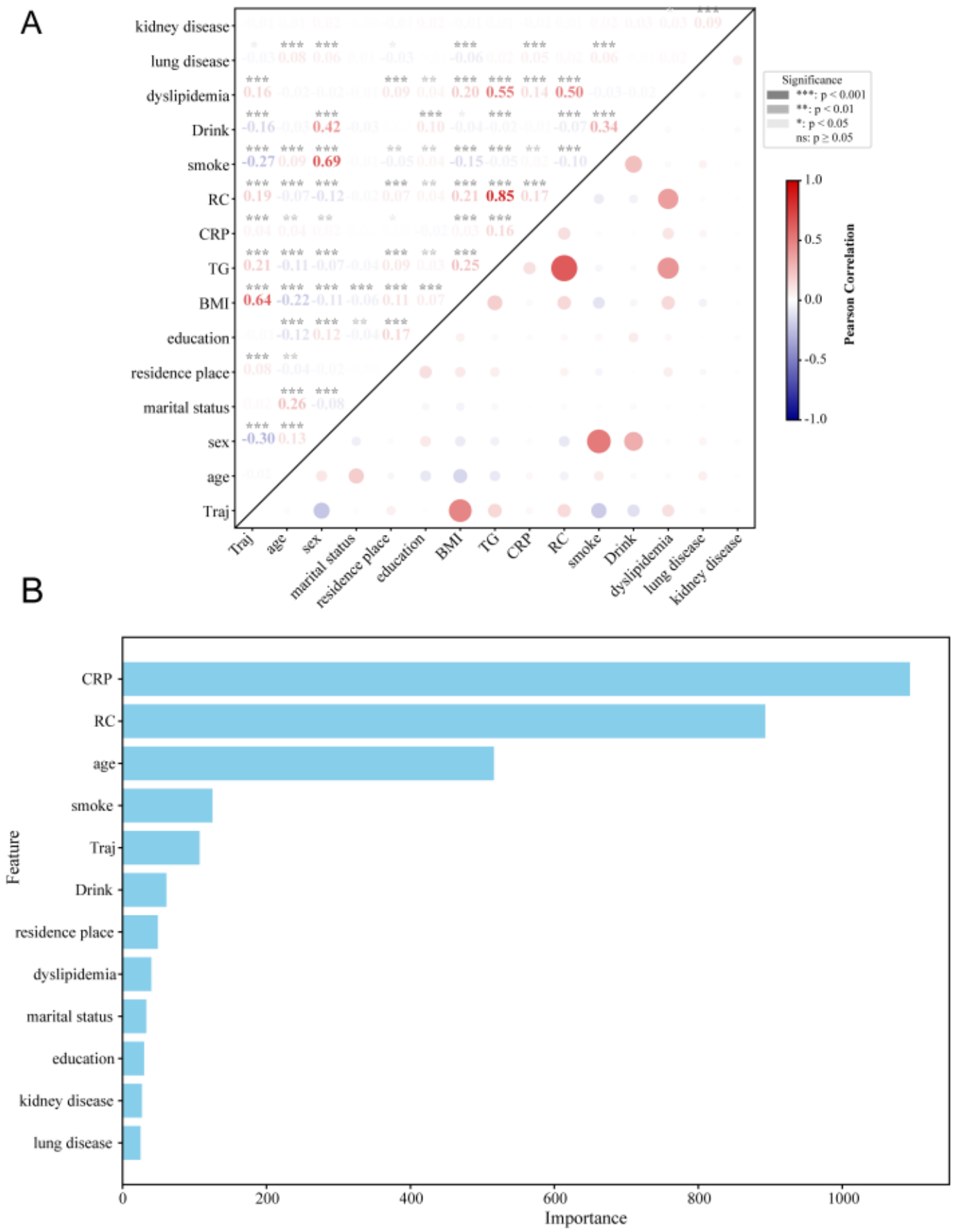

Figure S4 Feature selection and importance ranking for hypertension prediction using Light-GBM. (A) Correlation analysis among candidate variables was performed to identify and exclude highly collinear features prior to model development. (B) Feature importance ranking

based on Light-GBM identified the most informative predictors contributing to hypertension risk prediction.

Figure S5

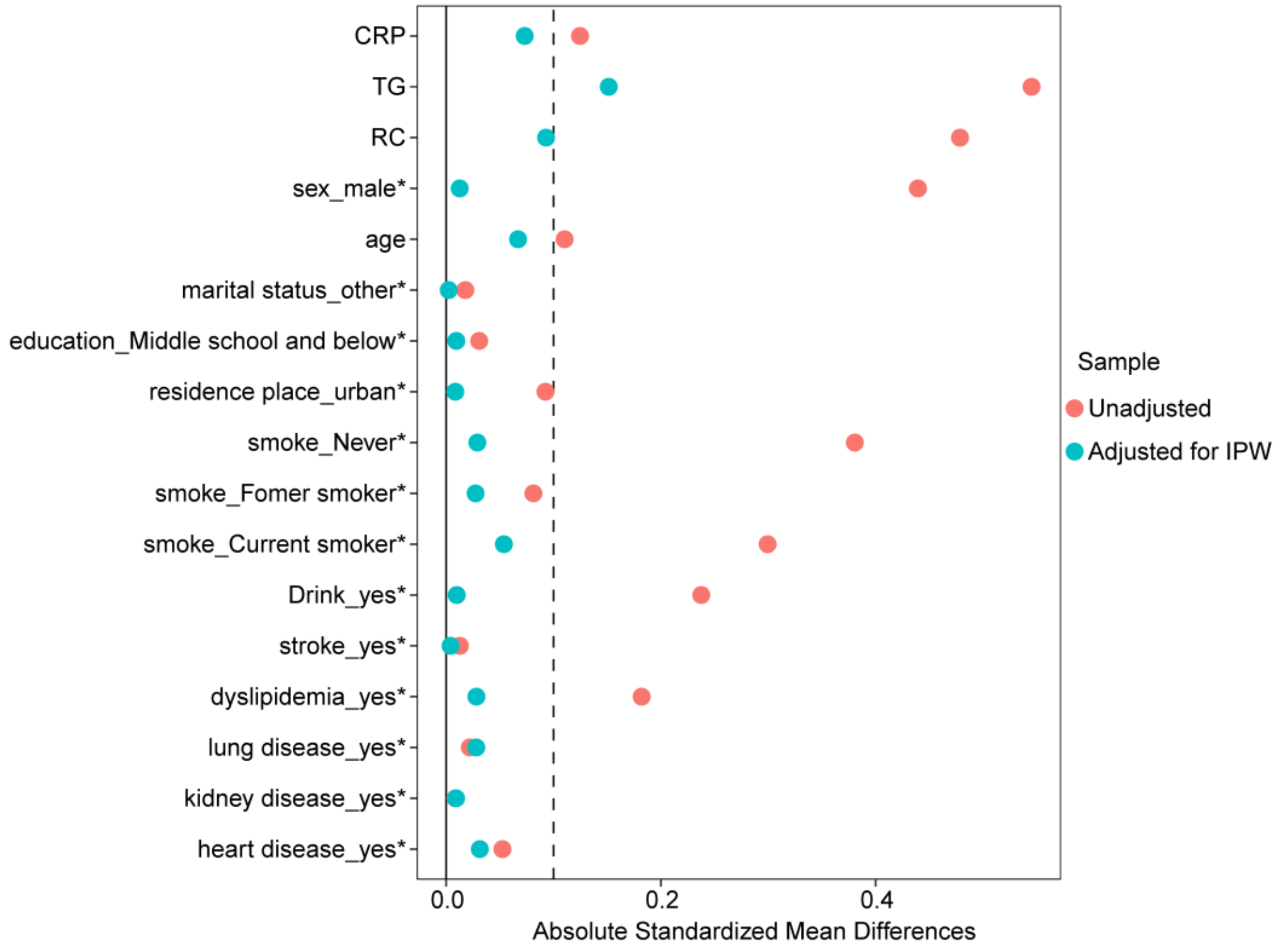

Figure S5 Differences in participant characteristics before and after weighting using the IPW method. Love plots show differences based on the original unweighted and inverse probability–weighted samples. IPW, inverse probability weighting.
